# Albumin-Based Hydrogel Films Covalently Cross-Linked with Oxidized Gellan with Encapsulated Curcumin for Biomedical Applications

**DOI:** 10.3390/polym16121631

**Published:** 2024-06-08

**Authors:** Camelia Elena Tincu (Iurciuc), Oana Maria Daraba, Christine Jérôme, Marcel Popa, Lăcrămioara Ochiuz

**Affiliations:** 1 Department of Natural and Synthetic Polymers, Faculty of Chemical Engineering and Protection of the Environment, “Gheorghe Asachi” Technical University, 73 Prof. Dr. Docent Dimitrie Mangeron Street, 700050 Iasi, Romania; camelia_tincu83@yahoo.com; 2 Department of Pharmaceutical Technology, Faculty of Pharmacy, “Grigore T. Popa” University of Medicine and Pharmacy, 16 University Street, 700115 Iasi, Romania; ochiuzd@yahoo.com; 3 Faculty of Dental Medicine, “Apollonia” University, 11 Pacurari Street, 700355 Iasi, Romania; maria.mary2019@yahoo.com; 4 Center for Education and Research on Macromolecules, Complex and Entangled Systems from Atoms to Materials, University of Liège, 4000 Liège, Belgium; c.jerome@ulg.ac.be; 5 Academy of Romanian Scientists, 3 Ilfov Street, Sector 5, 050044 Bucureşti, Romania

**Keywords:** albumin-based hydrogel, oxidized gellan, cross-linking of bovine serum albumin, biopolymers

## Abstract

Bovine serum albumin (BSA) hydrogels are non-immunogenic, low-cost, biocompatible, and biodegradable. In order to avoid toxic cross-linking agents, gellan was oxidized with NaIO_4_ to obtain new functional groups like dialdehydes for protein-based hydrogel cross-linking. The formed dialdehyde groups were highlighted with FT-IR and NMR spectroscopy. This paper aims to investigate hydrogel films for biomedical applications obtained by cross-linking BSA with oxidized gellan (OxG) containing immobilized β-cyclodextrin–curcumin inclusion complex (β-CD–Curc) The β-CD–Curc improved the bioavailability and solubility of Curc and was prepared at a molar ratio of 2:1. The film’s structure and morphology were evaluated using FT-IR spectroscopy and SEM. The swelling degree (Q%) values of hydrogel films depend on hydrophilicity and pH, with higher values at pH = 7.4. Additionally, the conversion index of -NH_2_ groups into Schiff bases increases with an increase in OxG amount. The polymeric matrix provides protection for Curc, is non-cytotoxic, and enhances antioxidant activity. At pH = 5.5, the skin permeability and release efficiency of encapsulated curcumin were higher than at pH = 7.4 because of the interaction of free aldehyde and carboxylic groups from hydrogels with amine groups from proteins present in the skin membrane, resulting in a better film adhesion and more efficient curcumin release.

## 1. Introduction

The skin is the body’s largest organ, composed of the epidermis, dermis, and hypodermis. Many people undergo invasive procedures to address skin defects but current approaches do not replicate the extracellular matrix’s microenvironment. Tissue engineering regenerates tissue defects using biomaterials to promote tissue and organ growth. Recent studies show promising results for skin tissue regeneration in diabetic and burn wound patients. Polymer-based therapeutic approaches for skin defects using scaffolds made of polymeric materials have become popular in recent years, promoting tissue regeneration [1]. When skin is injured, proper wound care plays a crucial role in avoiding bacterial infection and accelerating wound healing. The primary method is bandaging. Traditional wound dressings include gauze, cotton, and bandages, aiming to prevent the wound from external contamination [2].

However, they are not able to maintain a moist wound environment. As the exudate coagulates and the wound heals, the dressings adhere to the wound site, and the fibers are wrapped in new tissue or blood clots. Changing the dressing at regular intervals will damage newly formed tissues and cause pain to the patients, delaying the healing process [3].

Typical wound dressings only offer passive protection against external pollutants and do not actively enhance the healing process of wounds. Furthermore, these traditional wound dressings are ineffective in treating chronic wounds caused by infections and diabetes. Hence, there is a need for a new range of interactive dressings that provide physical protection and accelerate wound tissue regeneration to treat skin wounds [4,5,6] effectively. Advanced wound dressings can cover wounds and promote faster skin healing. One such type of dressing is hydrogels, which have recently gained attention. Hydrogels are three-dimensional cross-linked network systems composed of natural or synthetic polymers, with a water content ranging from 70% to 90% [7]. Hydrogels are unique materials that can be adjusted to mimic natural tissue’s composition and mechanical properties. They create enough space and mechanical support for cell migration and tissue regeneration.

Moreover, hydrogels can be engineered to have therapeutic agent-delivery capabilities, responsive properties, and smart-monitoring abilities, and can serve as delivery vehicles for therapeutic agents such as drugs, cells, and nanoparticles. Stimuli-responsive hydrogels can be used as smart, responsive materials to achieve the on-demand controlled release of loaded active substances [1,8]. The structure and composition of hydrogels closely resemble that of the natural extracellular matrix (ECM). These dressings provide a moist environment for the wound interface and have good absorption capabilities for tissue exudates. Additionally, by introducing functional groups or loading drugs, hydrogels can achieve different functions [7].

Hydrogels used for wound dressings are derived from biodegradable materials, which can be natural or synthetic polymers, each with its own advantages and disadvantages. Synthetic polymers used as dressings have the advantage of having their physical and chemical properties very well controlled. However, a significant disadvantage is that the materials based on synthetic polymers are not recognized by cells and are less likely to be used alone. Synthetic polymers used as drug delivery systems for wound care have lower biodegradability compared to natural polymers. Examples of these synthetic polymers include polylactic acid (PLA), poly(glycolic acid) (PGA), poly(lactic-co-glycolic acid) (PLGA), polyhydroxybutyrate (PHB), polycaprolactone (PCL), and polyanhydrides, such as poly sebacic acid, poly amino acids, polyphosphates, and polyurethane [9].

Biopolymers like polysaccharides and proteins are natural, abundant, and easy to obtain. Due to their biocompatibility and degradability, they are widely used in skin tissue engineering [10]. Protein-based hydrogels can be developed with tunable biochemical and mechanical properties, unique biocompatibility, and adequate biodegradability [11,12]. Obtaining biocompatible hydrogels from collagen gelatin, elastin, fibrin, silk fibroin, and serum albumin, which are also considered biodegradable materials from natural sources, is feasible due to their well-defined sequences, accessibility, and ability to be isolated and purified from natural sources [13,14]. Collagen is a commonly used protein for creating biomaterials to regenerate connective tissue due to its hydrophilicity, biocompatibility, and biodegradability. Collagen hydrogels are flexible and have low antigenicity [15]. However, the mechanical properties of these materials and complicated and expensive purification and isolation processes prevent their widespread use. Silk fibroin, a biodegradable biomaterial with human tissue-like properties, has been used in various biomedical applications. However, the resultant hydrogels did not have sufficient mechanical strength and are contraindicated for patients with third-degree burns [16,17,18,19,20]. Due to their exceptional properties, serum albumins have increasingly been used to develop biocompatible biomaterials in tissue engineering. Albumin hydrogels are noteworthy due to their non-immunogenicity, low cost, biocompatibility, and biodegradability. Artificial grafts, xenografts, and allografts are crucial in tissue reconstruction/repair. However, the human body considers them foreign materials and triggers an immune response to rejection. Developing autologous materials is a promising strategy to avoid immune responses and achieve ideal clinical results. Patient-derived albumin hydrogels show high potential for personalized treatment [21,22].

Serum albumin, a globular protein secreted by the body, has several advantages that have drawn the attention of researchers. Albumin is non-toxic, non-immunogenic, highly biocompatible, and stable in water and diluted salt solutions. Albumin has a half-life of 19 days in the bloodstream. Due to its role in the body, which is to interact with lipophilic molecules like hormones, fatty acids, vitamins (C, D, folic acid), and minerals (copper, zinc, calcium), the drugs encapsulated in albumin-based nanoparticles can be maintained in the bloodstream for an extended period compared to free drugs. Albumin also stabilizes the blood pH and is responsible for 80% of the osmotic pressure of plasma [13,14,15,16,17,18,19,20,21,22,23,24,25].

Bovine serum albumin (BSA) has a molecular weight of 66,267 Da and comprises 585 amino acid residues. It contains 17 disulfide bridges, a free sulphydryl group, and several disulfide bonds. Additionally, it has free thiol groups that can be used for further functionalization [26]. Research carried out until now shows that various drugs, genes, peptides, vaccines, and antibodies can effectively bind to albumin. This protein can be successfully used to obtain delivery systems with the controlled and targeted release of the encapsulated bioactive compounds. The albumin-based delivery systems have a high drug loading capacity, good biocompatibility, and biodegradability [27]. Albumin contains three functional groups, -COOH, -NH_2_, and –SH, which allow the protein to be easily functionalized with different ligands and improve the targeted release of the encapsulated drugs [28].

Albumin hydrogels can be obtained using various physical and chemical methods. Physical methods like pH change or high-temperature preparation can easily and inexpensively prepare albumin hydrogels. However, the properties of these hydrogels depend on various factors such as protein concentration, solution pH, incubation time, and temperature. It is important to note that the heat-induced preparation method can cause irreversible damage to some of the protein binding sites, which can affect albumin bioactivity. Therefore, physical parameters such as temperature, duration, and pH must be carefully adjusted to obtain the desired albumin hydrogels for specific applications [22]. In a study conducted by Arabi SH et al. [29], physical albumin gels with desired properties were prepared by changing the pH and temperature. The effects of changing pH, temperature, and incubation time on the formation of gels were also observed. Different solutions of acids and bases with an ionic strength of 2 M were used to change the pH of the albumin solutions. The results showed that gels can be formed at very low pH values (≤1) or high pH values (≥10.6) at low temperatures (below the protein denaturation temperature 62 °C). These pH values used for hydrogel preparation are different from those reported by Baler K. et al. [30], who reported that albumin gels were obtained at a pH value between 3 and 4. The researchers observed that by changing the pH of the solution, the charge distribution in BSA also changed, leading to partial denaturation of the protein and the formation of electrostatic bonds inside the hydrogel. It was found that hydrogels prepared under mild conditions (pH = 7.2 and room temperature) had weaker mechanical strength than hydrogels obtained at acidic pH and temperatures higher than 62 °C [22,29,30]. Albumin hydrogels are typically prepared using covalent cross-linking, and researchers have invested significant effort in improving their mechanical properties. Zhao et al. [31] developed a new biodegradable ruthenium–albumin hydrogel using glutaric aldehyde as a cross-linking agent. However, this cross-linking agent is toxic and unsafe for some biological applications like in situ cell delivery [31]. Researchers have created a new hydrogel that uses natural and safe cross-linking agents to address this issue. These agents form intermolecular disulfide bonds of BSA, which avoids using chemical cross-linking agents like epichlorohydrin, formaldehyde, and glutaraldehyde. These natural cross-linking agents are safe and eco-friendly, making them ideal for biomedical applications. They combine a natural reducing agent called glutathione (GSH) with tetrakis(hydroxymethyl)phosphonium sulfate (THPS), an environmentally friendly cross-linking agent that contains hydroxymethyl groups. These groups react with the primary and secondary amines of proteins. The researchers prepared albumin-based double-crosslinked hydrogels with dynamic covalent bonds [26]. J. Ouyang et al. [32] have developed a method for producing protein hydrogels without antibiotics, using THPS as a cross-linking agent. These hydrogels have superior biocompatibility and antibacterial properties, particularly against drug-resistant bacteria and biofilms. THPS stabilizes the hydrogel network and functions as an antimicrobial agent. Additionally, GSH acts as the primary cross-linking agent without requiring removal [32]. The formation of hydrogels was effective for at least 10 different proteins, improving the healing of wounds infected with drug-resistant bacteria.

In order to avoid the usage of toxic cross-linking agents, a different approach for obtaining protein-based hydrogels is to use oxidized polysaccharides to create new functional groups such as aldehydes. These aldehydes can then covalently interact with free amino groups in proteins and form imine or Schiff bases. Polysaccharide derivatives containing aldehyde groups are effective protein cross-linking agents [33]. Alginate dialdehyde is obtained by oxidizing alginate with NaIO_4_ [34]. The borate–diol complex formed binds gelatin through a condensation reaction, forming imine bonds, also known as Schiff bases [35]. This results in the development of an in situ injectable hydrogel that is biodegradable and provides sufficient anti-inflammatory or antioxidant stress responses. These cross-linked gelatin hydrogels allow cells to attach and migrate, facilitating osteoarthritis treatment. Another study found that the degree of cross-linking between gelatin and oxidized cellulose nanowhiskers increased with the number of aldehydes [36].

Gellan is an anionic extracellular polysaccharide secreted by the bacterium *Sphingomonas elodea*. The structural unit in gellan is composed of d-glucose, d-glucuronic acid, d-glucose, and l-rhamnose. It is a biocompatible FDA-approved food additive with favorable physical properties used in the food and pharmaceutical industries. Gellan hydrogels can be easily prepared and have been shown to be effective for various applications. Research has demonstrated that gellan-based hydrogels can maintain high cell viability and proper functionalities, suggesting that gellan has excellent potential for tissue engineering use [37,38].

In this paper, we will oxidize gellan using sodium metaperiodate. The objective is to obtain dialdehyde groups and decrease the gelation temperature of gellan. Through the oxidation reaction of gellan with NaIO_4_, two dialdehyde groups will be obtained in each structural unit of gellan at the vicinal dihydroxyl groups from C2–C3 of the rhamnose residue [39].

The main goal of this study is to develop and characterize a hydrogel obtained from bovine serum albumin (BSA) through the cross-linking reaction of amino groups from the protein with aldehyde groups from oxidized gellan (OxG). This hydrogel contains an encapsulated inclusion complex of curcumin/cyclodextrin with various applications in the biomedical field. Until now, no research has been conducted on albumin hydrogels cross-linked with oxidized polysaccharides or using OxG as a cross-linking agent for albumin.

Curcumin is a natural compound extracted from *Curcuma longa*, and recent clinical studies have shown its potential in cancer prevention and treatment. Researchers have found that oral administration of up to 12 g/day of curcumin is safe, suggesting low toxicity at the effective dose. Curcumin has been shown to regulate several important signal transduction pathways in survival, carcinogenesis, and apoptosis [40,41,42] and has been shown to have therapeutic effects against various forms of cancer and a strong anti-inflammatory effect. It reduces pro-inflammatory mediators such as lipoxygenase, cyclooxygenase-2, and inducible nitric oxide synthase [43] while inhibiting the expression of many inflammatory cytokines, such as TNF, IL-1, IL-6, and IL-8. Additionally, curcumin has been found to induce the expression and production of IL-10, which is an anti-inflammatory and immunosuppressive cytokine [44]. It has also been hypothesized that curcumin suppresses NF-κB, a transcription factor that induces the expression of various pro-inflammatory genes [45]. Curcumin is a compound that has many potential health benefits. However, when administered orally, it has low bioavailability due to poor absorption, rapid metabolism, and low water solubility. To increase its effectiveness, researchers are exploring new ways to improve its bioavailability, such as incorporating it into polymer-based delivery systems like micelles, liposomes, nanoparticles, hydrogels, inclusion complexes, and emulsions.

Additionally, combining curcumin with piperine can enhance its absorption. With its anti-inflammatory properties, low cost, favorable safety profile, and ease of use, curcumin is a promising therapeutic option for treating dermatological conditions [46]. Curcumin is known to be insoluble in water. Therefore, to increase its solubility, we obtained inclusion complexes of curcumin with β-cyclodextrin in a molar report of 2:1. These complexes were then immobilized in hydrogel films made of albumin and OxG. The resulting hydrogel films have the potential to be used for treating or improving certain dermatological conditions that have been mentioned in the literature [46].

The OxG obtained in this research paper was analyzed using FT-IR and ^1^H-NMR. Different oxidation times were used to determine the oxidation degree and molecular weight. The ability of aldehyde groups within OxG to cross-link the free amino groups within albumin was also analyzed by determining the conversion index of amine groups to Schiff bases by using different molar ratios between aldehyde and amine groups to obtain the hydrogel films. The hydrogels obtained with and without the curcumin/cyclodextrin inclusion complex were characterized using FT-IR spectroscopy, scanning electron microscopy, and swelling degree. The protective role of the polymer matrix was tested by evaluating the antioxidant activity of curcumin-containing films exposed to UV light for 30 min. The cytotoxicity of the hydrogel films was assessed on fibroblast cells, and the ability of the hydrogel films to control and sustain the release of curcumin was evaluated in vitro in buffer solutions on chicken skin used as a membrane for Franz cells in two different pH environments (7.4 and 5.5).

## 2. Materials and Methods

### 2.1. Materials

Gellan was purchased from Kelkogel (Atlanta, GA, USA); bovine serum albumin, β-cyclodextrin, tetraethylene glycol, sodium meta periodate NaIO_4_, sodium thiosulfate, ninhydrin, dimethyl sulfoxide (DMSO), and 2,2-diphenyl-1-picrylhydrazyl (DPPH) were purchased from Sigma-Aldrich (Burlington, MA, USA); and ethanol, sodium phosphate monobasic, sodium phosphate dibasic, sodium acetate, and acetic acid were purchased from Chemical Company (Iasi, Romania).

### 2.2. Methods

#### 2.2.1. Methods for Obtaining Albumin-Based Hydrogel Precursors and Films with or without Encapsulated Curcumin–β-Cyclodextrin Inclusion Complexes

- Obtaining Oxidized Gellan

One gram of gellan was dissolved in 100 mL of bi-distilled water at a temperature of 85 °C. The gellan solution was then cooled down to 35 °C. Next, 0.5 g of NaIO_4_ was dissolved in 20 mL of bi-distilled water, and the resulting mixture was added dropwise to the gellan solution while stirring. The reaction was carried out in the dark at 35 °C for the first 2 h and then at room temperature for 72 h. Different reaction times were used to determine the oxidation kinetics. After completion of the reaction, the product was dialyzed for 24 h using a dialysis membrane with a molecular weight cut-off of 14,000 kDa in distilled water to remove unreacted NaIO_4_. The water was changed at least five times during the dialysis process. After the dialysis, the product was freeze-dried and stored in a desiccator until further use.

- Obtaining the Inclusion Complexes of β-Cyclodextrin with Curcumin

The working method was adapted from Tang et al., 2002 [47]. In order to obtain inclusion complexes, a molar ratio of 2:1 between curcumin and β-cyclodextrin was used. Firstly, 3.4 g of β-cyclodextrin was dissolved in 50 mL of bi-distilled water at 60 °C, while 0.55 g of curcumin was dissolved in 20 mL of 98% ethanol. The curcumin solution was then added dropwise, using a dropping funnel, to the β-cyclodextrin solution while stirring at 500 rpm. The reaction mixture was stirred at 500 rpm and heated at 80 °C for 4 h under reflux. It was then kept at the same temperature for an additional hour to allow for the removal of ethanol while permitting air to enter the reaction environment. The cyclodextrin–curcumin inclusion complex was then kept in an ultrasonic bath at 40 °C for complete ethanol removal, which took about 2–3 h and left overnight under stirring at room temperature. The obtained product was kept for 12 h at 4 °C and filtered. The crystalline product obtained was dried in an oven at a temperature of 50–55 °C. After the inclusion complex was prepared, the complexation degree was determined by dissolving a certain amount of curcumin–β-cyclodextrin inclusion complex weighed before in 10 mL ethanol 98%. The amount of polyphenol within the complex was determined based on the calibration curve of curcumin in ethanol. The degree of complexation (CD, %) was determined as follows:

CD% = Amount of curcumin in the inclusion complex/total amount of curcumin × 100(1)

**Figure 1:**
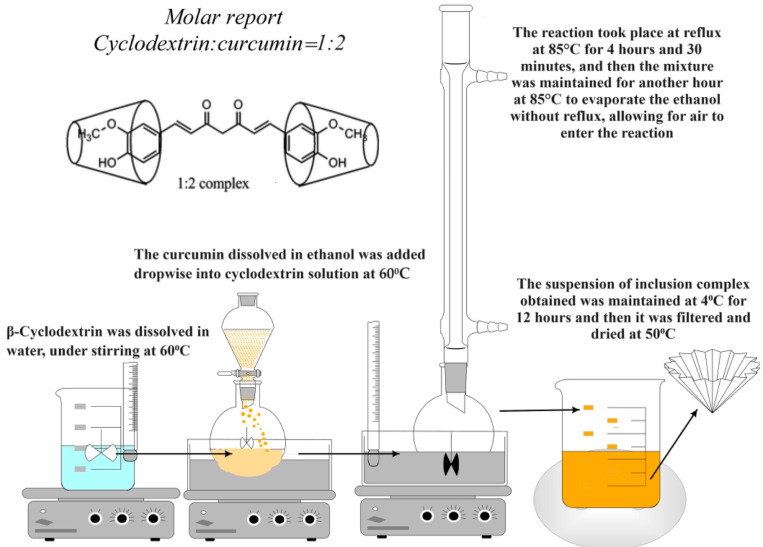
The schematization of the method used for obtaining the inclusion complexes of curcumin with β-cyclodextrin.

The calibration curve of curcumin in ethanol has the equation y = 0.0167x (R^2^ = 0.999). Figure 1 illustrates the process of obtaining β-cyclodextrin complexes with curcumin.

- Obtaining Hydrogel Films Based on Bovine Serum Albumin and Oxidized Gellan with Immobilized Inclusion Complexes

Various molar ratios were used between the amine groups in BSA and the aldehyde groups in OxG to produce hydrogel films. The quantity of BSA used remained constant, while only the amount of OxG was changed. In order to prepare the albumin solution, 0.1 g of BSA was dissolved in 2 mL of solution at pH = 3, 0.1 M, under stirring at 300 rpm and room temperature. The OxG was dissolved in 3 mL solution at pH = 3 at a temperature of 60 °C. After the OxG solution was cooled to room temperature, it was added dropwise to the BSA solution under stirring at 300 rpm.

**Figure 2:**
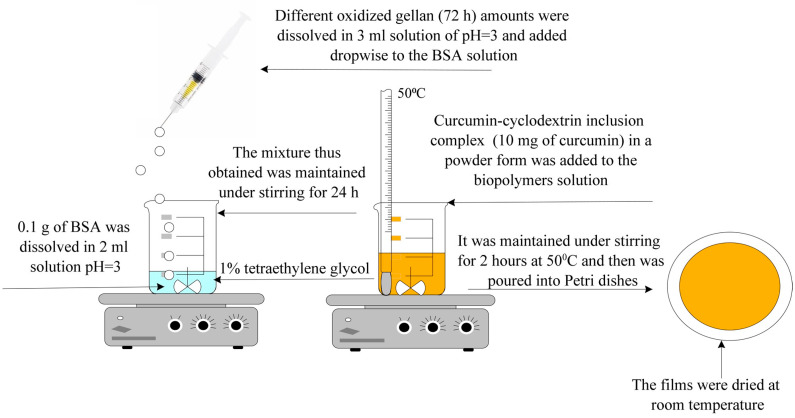
The preparation of the hydrogel films containing the curcumin–β-cyclodextrin complex immobilized.

A plasticizer of 1% (*w*/*w*) tetraethylene glycol was then added to the reaction. The reaction mixture (BSA, OxG tetraethylene glycol) was maintained under stirring (300 rpm) at room temperature for 24 h before being poured into Petri dishes and was left to dry at room temperature. After 48 h, the hydrogel films were removed from the Petri dishes and maintained on a filter paper until all water was evaporated and their weight remained constant. In order to obtain the hydrogel films that contained the encapsulated β-cyclodextrin–curcumin inclusion complex, we followed these steps: First, the reaction mixture was heated to 50 °C after 24 h, during which the cross-linking reaction between the NH_2_ groups of BSA and the aldehyde groups of OG occurred. Then, the inclusion complex, in powder form and containing 10 mg of curcumin, was added to the mixture under stirring and kept at 50 °C for 2 h until the entire inclusion complex amount was homogenized. As mentioned earlier, the resulting mixture was poured into Petri dishes and allowed to dry at room temperature. After drying, the films were removed from the Petri dishes and placed on filter paper until all the water had evaporated. The films were then weighed until their weight remained constant. The films were kept in a refrigerator at a temperature of 4 °C until further characterization. The obtaining method is schematized in Figure 2:

#### 2.2.2. Methods for Oxidized Gellan Characterization

- Kinetics of the Oxidation Reaction and Quantitative Determination of Aldehyde Groups Obtained from the Oxidation of Gellan with NaIO_4_

The aldehyde groups’ content was indirectly determined by titration of residual periodate dosed into the reaction mixture with iodometric titration, as described in Ref. [48]. After different oxidation times, a sample of OxG (1 mL) was taken and mixed with 1 mL of 20% KI and 1 mL of 37% HCl in an Erlenmeyer beaker. Iodine (I_2_) was titrated with 0.05 N Na_2_S_2_O_3_ until the color turned light yellow. Then, soluble starch was added to turn the titration solution blue. The titration was continued until the solution became transparent and iodine was no longer present in the reaction. Lange described these reactions in 1961 [49].

- KIO_4_ + 7KI + 8HCl → 8KCl + 4I_2_ + 4H_2_O
- I_2_ + 2Na_2_S_2_O_3_ × 5H_2_O → 2NaI + Na_2_S_4_O_6_

During the titration reaction, it was possible to determine the amount of NaIO_4_ that did not react. By subtracting this value from the total amount of NaIO_4_ used, we determined the amount of NaIO_4_ that reacted (as we know that eight moles of Na_2_S_2_O_3_ consume one mole of NaIO_4_). Notably, one mole of NaIO_4_ consumed is required to obtain two moles of aldehyde groups. Therefore, once the total amount of NaIO_4_ consumed in the oxidation reaction was established, we calculated the number of moles of aldehyde groups obtained after the oxidation reaction.

The working method for the study of oxidation kinetics:

First, the OG solution was prepared. In a flask of 250 mL, 1.5 g of gellan was added, which was dissolved in 150 mL of bi-distilled water at 85 °C. Once the temperature of the gellan solution decreased to 35 °C, 0.75 g of NaIO_4_ was added to the solution. The NaIO_4_ was dissolved in 30 mL of water and added dropwise while stirring at 300 rpm. As mentioned earlier, the reaction took place in the dark, and the flask was covered. To determine the oxidation kinetics (OD%), 1 mL of the sample from the reaction medium was taken from time to time (until 130 h), and it was titrated with Na_2_S_2_O_3_ as previously described; the degree of oxidation was calculated using the following equation:

(2) $$OD\%=\frac{Q_{R}}{Q_{T}}\times 100$$

where Q_R_ was the total amount of NaIO_4_ that reacted, and QT was the total amount of NaIO_4_ that could respond, as determined stoichiometrically (in the case of OxG, it is 0.495 g).

- Determination of the Molecular Weight of Oxidized Gellan

The intrinsic viscosities of gellan and OxG were measured by working with various concentrations of gellan or OxG (0.1%, 0.2%, 0.25%, 0.3%, and 0.4% *w*/*v*) in a 0.1 M acetate buffer solution with a pH of 3.5. The measurement was conducted using a Ubbelohde viscometer (Schott, Hofheim, Germany) at 23 °C. The molecular weight (Mw) was calculated using the Kuhn–Mark–Houwink equation.

[η] = K × Mw^α^ (3)

where K = 7.49 × 10^−3^ și α = 0.91 [39], [η] represents the intrinsic viscosity, and Mw is the molecular weight.

- FT-IR Spectroscopy for Gellan and Oxidized Gellan

FT-IR spectra were recorded for gellan and OxG. The oxidation reaction took place for 72 h. The spectra were recorded using an FT-IR Cary 630 (Agilent Technologies, Santa Clara, CA, USA) with ATR at the sample interface. The frequency range for recording was between 4000 and 650 cm^−1^, with a resolution of 4 cm^−1^.

- Nuclear Magnetic Resonance Spectroscopy

Nuclear magnetic resonance spectroscopy (Bruker 300 RMN) was employed to investigate the gellan’s molecular structure before and after oxidation. The samples were prepared by dissolving gellan or OxG for 72 h in deuterated water at 80 °C, then analyzed using the ^1^H-NMR technique.

#### 2.2.3. Hydrogel Film Characterization Methods

- Determination of Albumin’s Free Amine Groups Using Ninhydrin Assay and Evaluation of Conversion Index of Hydrogel Films

The number of free amino groups in BSA was determined using the ninhydrin assay. Firstly, a calibration curve of glutamic acid concentration (or the number of moles of amino groups) vs. absorbance was plotted. Then, the free amino groups in albumin were determined using the ninhydrine test. In order to do this, a stock solution of BSA was prepared in a 0.1 M acetate buffer solution with a pH of 5.6 and a concentration of 0.1%. Different volumes were taken from this solution to obtain albumin solutions of varying concentrations (between 100 µg/mL and 500 µg/mL). For the determination of the free amino groups in a BSA solution, 1 mL of the sample (which had different concentrations) was added to a test tube. Then, 1 mL of ninhydrin solution ethanol with a concentration of 0.2% was added over the sample in the test tube and heated at 95 °C for 30 min. After the solution was cooled, 8 mL of ethanol:distilled water solution (1:1 volume ratio) was added to the test tubes.

We measured the optical absorbance of the solutions using a UV spectrophotometer at 570 nm wavelength. The number of moles of free amino groups in the BSA was calculated using the calibration curve of glutamic acid that was previously plotted. We followed the same procedure to determine the absorbance of glutamic acid solutions with known concentrations to plot the calibration curve. The results were expressed as moles of NH_2_ groups per gram of BSA. The equation of the calibration curve of glutamic acid obtained was 10^7^y = 0.2241x for the number of moles of NH_2_ groups vs. absorbance with an (R^2^ = 0.9903).

The obtained hydrogel films were tested to determine the conversion index of amino groups into Schiff bases. The films were weighted with varying molar ratios between the number of free amine groups in BSA and the number of aldehyde groups in OxG. Before the test, a small piece of each film type was also weighed. The film fragment was added to a test tube containing 1 mL of 0.1 M acetate buffer solution (pH = 5.6) and 1 mL of 0.2% ninhydrin solution. The above protocol was also used to determine the free amine groups in BSA-based hydrogels. The difference between the total number of amino groups initially added into the hydrogel film and the free amino groups determined with the ninhydrin test within the hydrogel gives the number of linked amino groups. The amount of unbound amino groups in BSA was measured through optical absorbance before ($C_{b}$) and after ($C_{a}$) cross-linking. The extent to which amine groups reacted with carbonyl groups in the films to form Schiff bases was determined by calculating a conversion index (CI%)-relation (4). All the determinations were made in triplicate, and the results were presented as average values ± STDEV.

(4) $$CI\%=\frac{C_{b}-C_{a}}{C_{b}}\times 100$$

- Determination of Encapsulation Efficiency

First, a calibration curve was constructed for curcumin in ethanol using known concentrations of curcumin solutions between 5 and 60 μg/mL. The optical absorbance of these solutions was determined using a nanodrop spectrophotometer at the wavelength of 425 nm. The calibration curve equation is y = 0.0167x (R^2^ = 0.999).

In order to determine the amount of curcumin encapsulated in the hydrogel films, the following method was used: First, the dry fragments of the hydrogel film containing the curcumin–β-cyclodextrin inclusion complex from the samples listed in Table 1 were weighed and then immersed in 15 mL of ethanol in Erlenmeyer beakers in the dark using a water bath. For curcumin extraction, the hydrogel films with encapsulated curcumin immersed in ethanol were maintained under stirring for 24 h at room temperature and after the quantity of curcumin was determined spectrophotometrically at 425 nm using the previously plotted calibration curve. In order to ensure that all the curcumin was extracted, the extraction process continued for an additional 6 h, during which the concentration of curcumin was determined again. The fact that there were no differences between the two concentration values led to the conclusion that the polyphenol was completely extracted. Finally, the encapsulation efficiency (Ei%) expressed as a percentage was calculated using the following formula:

E_i_ (%) = M_cf_/M_i_ × 100 (5)

where M_cf_ is the spectrophotometrically determined curcumin amount in the film, and M_i_ is the initial curcumin amount added to the film. Three determinations were performed for each sample, and results are presented as mean values ± STDEV.

- FT-IR Spectroscopy of Hydrogel Films with Immobilized Curcumin

Fourier Transform Infrared (FT-IR) spectra were collected for samples, including BSA, OxG for 72 h, and the β-cyclodextrin–curcumin inclusion complex. The spectra were also obtained for hydrogel films with and without incorporated curcumin. The measurements were recorded at the sample interface using an FT-IR Cary 630 instrument (Agilent Technologies) with Attenuated Total Reflection (ATR) in a frequency range from 4000 to 650 cm^−1^ and a resolution of 4 cm^−1^.

- Scanning Electron Microscopy (SEM)

Hydrogel films based on BSA and OxG were analyzed by scanning electron microscopy (SEM) to determine their morphology and demonstrate that curcumin was immobilized in the matrix of BSA cross-linked with OxG. The hydrogel films were dried, sputter-coated with gold, and analyzed using a Vega Tescan instrument (Tescan, Kohoutovice, Czech Republic).

- Determination of the Ability of Hydrogel Films to Retain Water

The films obtained have a hydrogel-like nature, making it important to determine their water retention ability through the swelling degree (Q, %). This feature is crucial because the swelling degree values affect the intensity of curcumin diffusion from the hydrogel matrix. Gravimetric analysis was used to determine the Q (%) values of the hydrogel films obtained. Two aqueous solutions of different pH values were used to simulate physiological pH: 0.1 M phosphate buffer solution (PBS) at pH 7.4 (simulating blood pH) and acetate buffer solution (ABS) at pH 5.5 (simulating normal skin pH).

An accurately weighted amount of dry film (Mdry) was placed in 10 mL of a swelling agent at 37 °C. Periodically, the swelling agent was filtered, and excess liquid was removed by blotting the film’s surface with filter paper. The weight of the swollen film (M_swollen_) was determined by weighing it on an analytical balance. The amount of swelling agent retained by the hydrogel film (M_swelling agent_) was calculated by subtracting the weight of the dry film (M_dry_) from the weight of the swollen film (M_swollen_). The samples were then re-immersed in the swelling agent (always 10 mL), and the process was repeated until equilibrium was achieved. The swelling degree was expressed as the ratio of the amount of swelling agent present in the films at each time interval to the amount of completely dry film (Equation (6)). Three determinations were performed for each sample, and the results are presented as mean values ± STDEV.

(6) $$Q(\%)=\frac{M_{\mathrm{sweelling}\;\mathrm{agent}}}{M_{\mathrm{dry}}}\times \;100$$

- Determination of Antioxidant Activity

This method, with some modifications, was previously described by Mensor et al. [50] and Choi et al. [51]. In order to prepare the curcumin stock solution, 10 mg of curcumin was dissolved in 50 mL of ethanol. Dilutions were made to achieve final concentrations of 10, 15, 20, 25, 30, 35, 40, and 50 μg/mL. In the experiment, 2 mL of each concentration of curcumin was added to the test tubes. Then, 2 mL of DPPH solution in ethanol with a concentration of 0.1 mM was added to the curcumin solution in the test tubes. The samples were then vortexed for 20–30 s. After 40 min, the absorbance of the samples was read using a UV spectrophotometer at a wavelength of 517 nm. Ascorbic acid was used as the standard. The absorbance values were then converted into antioxidant activity percentage, also known as DPPH free radical inhibition percentage, using the following Formula (7):

(7) $$I\%=100-[\left(A_{s}-A_{b}\right)\times \frac{100}{A_{c}}]$$

The IC50 value represents the concentration of curcumin required to capture 50% of free radicals from DPPH, as calculated from the plot of I% versus concentration. The spectrophotometer was calibrated using ethanol. The absorbance values of the solutions containing 2 mL of DPPH solution and different sample concentrations were represented by “A_s_”. A blank solution (A_b_) was prepared using ethanol (2 mL) and curcumin solutions of various concentrations (2 mL), and its absorbance value was measured for each concentration. The control solution (A_c_) was prepared using 0.1 mM DPPH solution (2 mL) and ethanol (2 mL). The determinations were performed at room temperature in triplicate. In order to test the antioxidant activity of the films based on BSA/OxG containing curcumin, the samples were irradiated with UVA at 365 nm for 30 min and non-UV irradiated. To extract the curcumin from the hydrogel films, a known amount of hydrogel film was immersed in 25 mL of ethanol and kept under stirring in Erlenmeyer beakers in a water bath at a physiological temperature of 37 °C for 24 h in closed containers in the dark. After extraction, the curcumin concentrations of the samples in 25 mL ethanol were determined spectrophotometrically at 425 nm using the calibration curve of curcumin in ethanol. Thus, the curcumin stock solutions were obtained for each type of hydrogel film with different NH_2_/CHO molar ratios, and different dilutions were made to obtain concentrations of 10, 15, 20, 25, 30, 35, 40, and 50 μg/mL, which are the same as those of free curcumin. The work mode for antioxidant activity determination was the same as in the case of free curcumin. The percentage of DPPH free radical inhibition was then determined, and based on these results, the IC50 was calculated for both the curcumin extracted from the UV-irradiated hydrogel films and the curcumin extracted from the non-irradiated films. The ascorbic acid was used as standard. All measurements were made in triplicate to ensure accuracy.

- Cytotoxicity Evaluation of Hydrogel Films without Curcumin

In order to evaluate the potentially harmful effects of hydrogel films based on BSA and OxG on cells, adult human dermal fibroblast cells (HDFa) were used. The viability of these cells was tested after incubation with hydrogels for 24 and 48 h. A colorimetric method was used to determine cell viability, which involved the use of 3-(4,5-dimethylthiazol-2-yl)-2,5-diphenyltetrazolium bromide (MTT). Each material was tested twice, and the results were compared to a blank. Hydrogels without the inclusion complexes of curcumin–β-cyclodextrin immobilized A2, A4, and A7 were tested. The materials were sterilized using UV–Vis radiation for 3 min and then placed in direct contact with fibroblast cells cultured in 96-well plates. The cells were grown using DMEM (Dulbecco’s Modified Eagle Medium) growth medium supplemented with 10% FBS (Fetal Bovine Serum), 1% (*v*/*v*) penicillin-streptomycin, and 1% (*v*/*v*) non-essential amino acids. This medium was used to promote cell proliferation. After 24 and 48 h of incubation, the samples were analyzed using a Multiskan FC automated microplate reader (Thermo Fisher Scientific Inc., Waltham, MA, USA) at λ = 570 nm. Microscopic analysis was carried out with an inverted microscope CKX41, OLYMPUS, Tokyo, Japan.

- Release Kinetics of Curcumin from Hydrogel Films

This study examined the release kinetics of curcumin in two different pH media—in a 0.1 M phosphate buffer solution at pH = 7.4 (which is the specific pH of blood) and in a 0.1 M acetate buffer solution at pH = 5.5 (which is specific to pH of skin) at 37 °C.

In order to investigate the release of curcumin within the hydrogel films when applied to the skin, a Franz cell was utilized. The cell had a diameter of 1.37 cm and a receptor compartment with a volume of 7.5 mL. The receptor compartment of the Franz cell was separated by a chicken skin membrane. This study was conducted in vitro.

The skin used for the experiment was degreased with 96% ethyl alcohol, and only the skin without defects or open pores was utilized. After preparation, the skin was maintained in a 10% glycerin solution for 24 h before use. The membrane was then clamped between the donor and receiver compartments, and films containing immobilized β-cyclodextrin–curcumin were placed on the skin membrane surface in the Franz cell donor compartment. Acetate buffer 0.1 M with a pH of 5.5 or phosphate buffer 0.1 M with a pH of 7.4 containing 1% (*w*/*w*) Tween 80 were used as release media. Finally, 1 mL of solution with pH 5.5 or 7.4 was placed in the donor container for the experiment. The release kinetics study of curcumin was conducted in the absence of light using a Franz cell covered with aluminum foil. The temperature was maintained constantly at 37 °C while stirring the mixture at 200 rpm. At specific intervals, 0.5 mL of the medium was removed from the receptor compartment to measure the quantity of released curcumin. The removed medium was then replaced with a fresh medium. The release profiles were obtained by measuring the concentration of curcumin using a nanodrop UV–Vis spectrophotometer at 425 nm. The permeability of curcumin (P) within the films through the skin membrane was calculated and expressed as μg curcumin/cm^2^·h after 24 h using Equation (8).

(8) $$P=\frac{M_{r}+M_{s}}{S},\;\frac{µg}{{cm}^{2}}$$

where M_r_ represents the amount of curcumin released in the receptor compartment, M_s_ represents the amount of curcumin within the skin, and S represents the film’s surface expressed in cm^2^. After 24 h, in which curcumin-released kinetics was studied, the polyphenol extraction within the skin membrane was made in dimethyl sulfoxide (DMSO) solution (15 mL) in the dark, under stirring, for 24 h at room temperature. The amount of curcumin within the skin membrane extracted in DMSO was determined spectrophotometrically based on the calibration curve of curcumin in DMSO (y = 0.0178x, R^2^ = 0.9997). In order to ensure that all the curcumin was extracted, the skin membrane was maintained in the DMSO solution for another 6 h, during which the concentration of curcumin was determined again. The fact that there were no differences between the two concentration values led to the conclusion that the polyphenol was completely extracted in DMSO.

In chemistry and biology, the permeability coefficient refers to the rate at which a substance moves through a permeable membrane. Equation (9) describes the permeability coefficient (P_c_):

(9) $$P_{c}=\frac{M_{r}+M_{s}}{S\times t},\;\frac{µg}{{cm}^{2}\times h}$$

where t represents the release time expressed in hours.

Finally, the release efficiency (%) was calculated using Equation (10).

(10) $$R_{Ef}\%=\frac{M_{r}+M_{s}}{M_{i}}\times 100$$

The amount of curcumin released was then determined at a wavelength of 425 nm. The equations of the calibration curves used to determine the curcumin released from the films were (λ = 425 nm).

- For calibration curve of curcumin in 0.1 M PBS at pH = 7.4: y = 0.0079x (R^2^ = 0.9983).
- For calibration curve of curcumin in 0.1 M in ABS at pH = 5.5: y = 0.0104x (R^2^ = 0.9991).

## 3. Results and Discussions

### 3.1. Obtaining Oxidized Gellan and Covalently Cross-Linked Albumin-Based Hydrogel Films Containing Cyclodextrin Inclusion Complex with Curcumin

**Figure 3:**
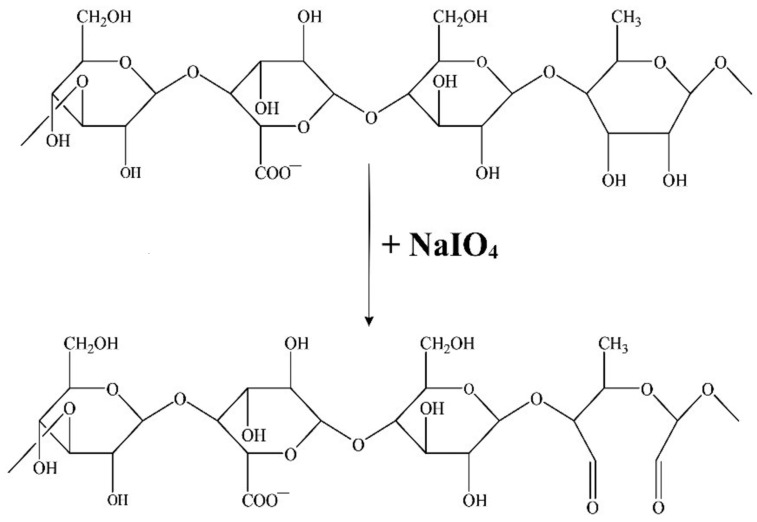
The structure of gellan and the schematization of the oxidation reaction of gellan in the presence of NaIO_4_.

Figure 3 presents the gellan’s structure and the oxidation reaction’s schematization.

Oxidation with periodate (IO_4_^−^) was used for polysaccharide structural analysis. The polysaccharide was oxidized in the presence of NaIO_4_, reducing its molecular mass, followed by mild acidic hydrolysis [52,53]. Oxidation of polysaccharides in the presence of sodium periodate is a well-known reaction that leads to the specific oxidation of free vicinal dihydroxyl groups from C2–C3 to aldehydes with cleavage of the internal ring, creating polyaldehydes [54]. The oxidation reaction with periodate is typically performed in aqueous systems. However, in such systems, the aldehyde groups of polysaccharides that are oxidized with periodate can react with hydroxyl groups, resulting in the formation of inter- or intra-hemiacetal linkages, or react with water, forming hemialdals or hydrated aldehydes [55]. Complicated saccharide structures can form from aldehyde oxidation side reactions, making the polysaccharide analysis after periodate oxidation difficult [56]. In this paper’s research, NaIO_4_ was used as an oxidizing agent to cleave the adjacent dihydroxyl groups from the polysaccharide chain, which leads to the hydrolysis of glycosidic bonds and the degradation of gellan. The oxidation reaction takes place in distilled water at pH = 7 in the dark and at room temperature using varying oxidation times, and using water or an aqueous solvent as a reaction medium is a characteristic of the oxidation of polysaccharides in the presence of sodium periodate. For most carbohydrates, water is an ideal reaction medium. The oxidation rate in ethanol–water and acetic acid in water is lower than in water. These media are effective in the oxidation reaction in the presence of sodium periodate, as they are a better means of decreasing the reaction rate than when low temperatures are used for this purpose [57]. According to Lindstedt [58], the oxidation of carbohydrates in neutral and acidic media led to similar results when the C–C bonds were cleaved, and no hydroxyaldehydes were formed. However, the results were higher than the expected values in a neutral solution. The researcher demonstrated that the mannan yeast molecule was almost fully oxidized to carbon dioxide and water in the presence of NaIO_4_ if the pH was above 5. Notably, the reaction temperature was raised to 50 °C, which is not recommended for working with sodium periodate [58]. Reducing the reaction temperature can help slow down the acetal’s hydrolysis rate, which is one of the main reasons for “over-oxidation”. In order to achieve maximum selective oxidation and minimum “over-oxidation”, it is recommended to carry out oxidations with periodate at the lowest possible temperature that is suitable for the used solvent system and the solubility of the reactants [57].

We selected water as the neutral medium to oxidize the polysaccharide with NaIO_4_ because gellan in an acidic pH forms a gel that is reversible with increasing temperature, and the solution becomes more viscous even at low polymer concentrations. When the pH is lowered to a pKa value of 3.5, the polymer chains in the junction areas interact more strongly with each other, resulting in a polysaccharide structure with fewer disordered chains. However, the network aggregates more when the pH is below the pKa value, resulting in phase separation between the solvent and polymer [59,60]. In our case, this would necessitate the use of higher oxidation temperatures. In an alkaline environment, two aldehyde molecules can condense to form aldols, and this medium causes overoxidation of gellan. Figure 3 displays the structure of gellan and the oxidation reaction. In the process of oxidizing gellan with NaIO_4_, two aldehyde groups are formed in every structural unit of gellan at the rhamnose residue. These aldehyde groups can react with the free amino groups present in BSA. This reaction leads to the formation of imine bonds (Schiff bases) and ultimately results in BSA-based hydrogels covalently cross-linked with aldehyde groups derived from OxG. To create hydrogels of BSA and OxG with and without the encapsulated curcumin–β-cyclodextrin inclusion complex, we followed the experimental program shown in Table 1.

**Figure 4:**
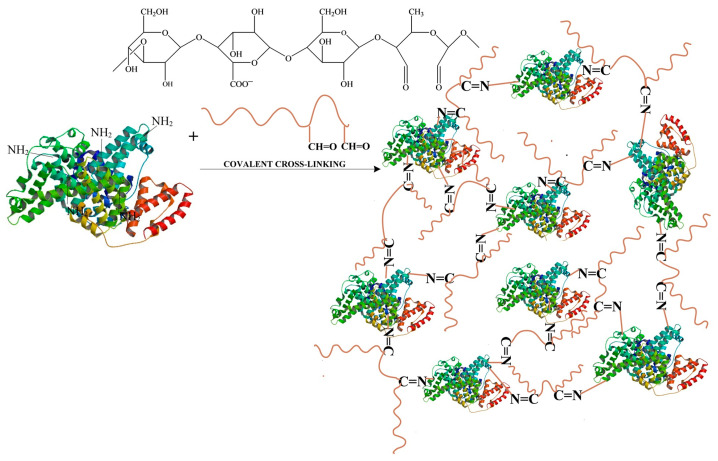
Schematic presentation of the cross-linking reaction between the amine groups from albumin and the aldehyde groups from the OxG.

Curcumin is a substance that does not dissolve in water and has a low bioavailability [61]. In order to increase the solubility of curcumin in water and, implicitly, its bioavailability [62], we chose to prepare an inclusion complex of β-cyclodextrin–curcumin, which will be included in the obtained films. Albumin films were obtained with OxG at a pH of 3. However, at higher pH values, albumin precipitated when OxG was added, probably because the carboxylic groups in gellan react with the amine groups in albumin to form polyelectrolyte complexes. The pKa value for gellan carboxylic groups is at pH = 3.5 [60]. At pH values ≥ 3.5, electrostatic interactions can occur. The isoelectric point of albumin, which is the pH value at which it has no electrical charge, is found between 4.6 and 4.9 [63]. At lower pH values, the amine groups within BSA become protonated and can react with the aldehyde groups within OxG. The formation of Schiff bases also depends on the reaction time, and the reaction between OxG and albumin takes place over a period of 24 h. Figure 4 schematically presents the structure of the albumin films obtained with OxG.

As mentioned before, curcumin has low bioavailability due to its water insolubility. Although it dissolves in polar organic solvents [63,64,65], the presence of organic solvents causes biopolymers to precipitate if we try to encapsulate curcumin in hydrogel films. In order to avoid this, it was decided to obtain a β-cyclodextrin–curcumin inclusion complex in a molar ratio of 2:1, which can then be included in hydrogel films based on BSA/OxG. Cyclodextrin inclusion complexes have an advantage over other materials as they contain a hydrophobic cavity that can encapsulate various lipophilic molecules. β-cyclodextrin is a cyclic oligosaccharide with seven glucose units linked by α-(1,4)-glycosidic bonds [66]. The molecular weight of hydrophobic compounds that can be included in the cavity of cyclodextrin ranges from 200 to 800 g/mol. Due to its availability and reasonable price, curcumin could be used to form inclusion complexes with β-cyclodextrin in a 2:1 molar ratio. In this ratio, the two phenolic rings of curcumin are encapsulated by cyclodextrin [67]. The degree of complexation of curcumin was determined after each synthesis and found to be between 50 and 55%. To prepare hydrogel films with encapsulated curcumin, β-cyclodextrin–curcumin inclusion complexes containing 10 mg of curcumin in powder form were added to the biopolymer solutions after the cross-linking reaction. The inclusion complex was observed to be solubilized in the biopolymer solution, indicating that curcumin could be immobilized in the biopolymer films.

### 3.2. Kinetics of the Oxidation Reaction and Quantitative Determination of Aldehyde Groups Resulting from Gellan Oxidation

**Figure 5:**
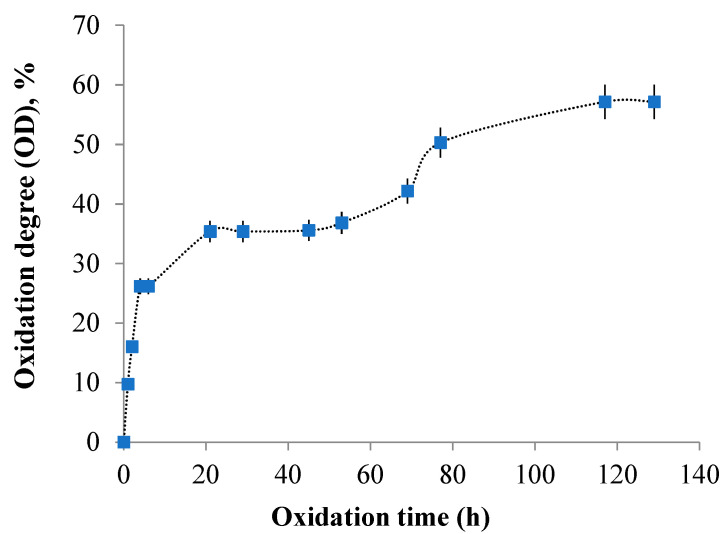
Oxidation kinetics of gellan in the presence of NaIO_4_.

The consumption of sodium meta periodate was measured to monitor the oxidation degree during the reaction between gellan and sodium periodate. Figure 5 displays the obtained results.

In order to determine the degree of oxidation, a 150 mL volume of 1% gellan solution was prepared. The amount of sodium periodate used was 0.75 g, which exceeded the stoichiometrically calculated amount by 52%. The total amount of NaIO_4_ that could react with 1.5 g of gellan, calculated stoichiometrically, was 0.495 g. By knowing the volume of sodium thiosulfate used in the titration, we can determine the amount of NaIO_4_ that did not react with the OxG. According to the Lange 1961 method, it takes eight moles of Na_2_S_2_O_3_ to consume one mole of NaIO_4_. By subtracting the initial amount of sodium periodate added from the total amount, we can calculate the amount of sodium periodate that reacted with the gellan. Based on Figure 5, the degree of oxidation of gellan gradually increased until 21 h. After this time, it remained constant until 48 h, then increased again until 117 h. The maximum degree of oxidation was 57.16%, but not all of the sodium meta periodate reacted, meaning that only a portion of the gellan was oxidized. It is well-known that an acidic environment promotes oxidation reactions but it can also cause unwanted side reactions, such as the formation of hemiacetals [68].

The average number of aldehyde groups per gram of OxG is 1.83 × 10^−3^ moles of -CHO/g after 72 h.

### 3.3. Determination of the Molecular Weight of Oxidized Gellan

**Figure 6:**
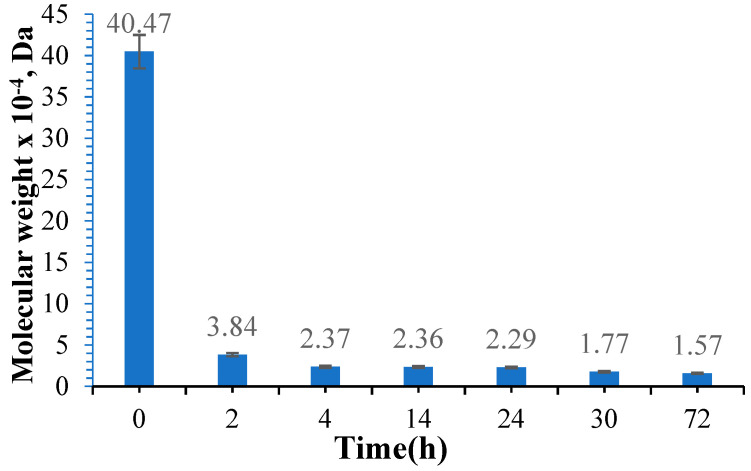
Variation of gellan molecular mass during the oxidation process.

Figure 6 presents the variation of the molecular weight of gellan in the oxidation reaction using different reaction times.

In order to determine the molecular mass, gellan was dissolved in an acetate buffer solution at pH = 3.5. The amount of sodium periodate, gellan concentration, and temperature were maintained constantly. Figure 6 shows that the molecular weight and intrinsic viscosity decrease as the oxidation time increases. Gong, Y. et al. [39] reported similar findings in 2009, except that the molecular weight of the standard gellan they obtained was approximately ten times higher than that of gellan from Kelkogel [39]. This difference may be attributed to the type of gellan used. In the study conducted by Dentini M et al. in 1988, they used the Mark–Hawkings equation with the same K and α constants (K = 7.49 × 10^−3^ and α = 0.91) and determined the molecular weight of 43.4 × 10^4^ Da for standard gellan [69], and in the work of Dreveton et al. [70], the molecular weight for different types of gellan ranged from 31 × 10^4^ Da to 62 × 10^4^ Da [70]. Similar to other studies on the oxidation of gellan, the intrinsic viscosity of OxG decreases as the oxidation time increases. However, previous research has demonstrated that the quantity of oxidizing agents also influences the molecular weight and intrinsic viscosity used [39,71]. The molecular weight decreases when the concentration of oxidizing agent increases. In our research article, our objective was to increase the oxidation degree of gellan in order to produce a higher number of aldehyde groups for cross-linking reactions with amino groups within BSA. In order to achieve this, we used an excess of NaIO_4_ as the oxidizing agent. Following oxidation, the OxG was used as a cross-linking agent for the amino groups in BSA, resulting in a hydrogel film with imine bonds (Schiff bases).

### 3.4. FT-IR Spectroscopy for Standard Gellan and Oxidized Gellan

**Figure 7:**
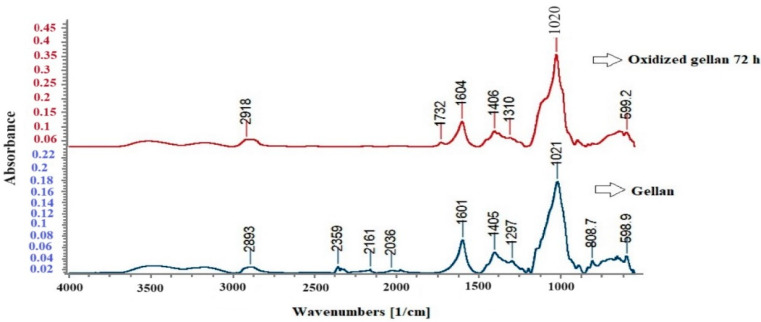
FT-IR spectra of the standard gellan and OxG after 72 h of oxidation.

Figure 7 shows the FT-IR spectra of standard gellan and OxG after 72 h.

Figure 7 shows that the standard gellan and the OxG obtained after 72 h of oxidation exhibit characteristic absorption peaks at approximately 2900, 1601, 1405, and 1021 cm^−1^. The band at around 2900 cm^−1^ corresponds to the C-H vibrations of the polysaccharide. The strong band at about 1020 cm^−1^ is specific to carboxylic groups in gellan [72]. The absorption peak at 1405 cm^−1^ can be assigned to either the -C-O- stretching vibration of the carboxylic group or the -CH_3_ group in the rhamnose residue, and it is found in both spectra. These corresponding peaks in the OxG spectrum show only slight changes. The results demonstrate that in the spectra of OxG, a new characteristic absorption peak appears at 1732 cm^−1^, which is between 1730 and 1740 cm^−1^, and is attributed to the -C=O group from the aldehyde groups. This absorption peak confirms that the oxidation reaction between gellan and NaIO_4_ occurred.

### 3.5. ^1^H-NMR Spectroscopy

**Figure 8:**
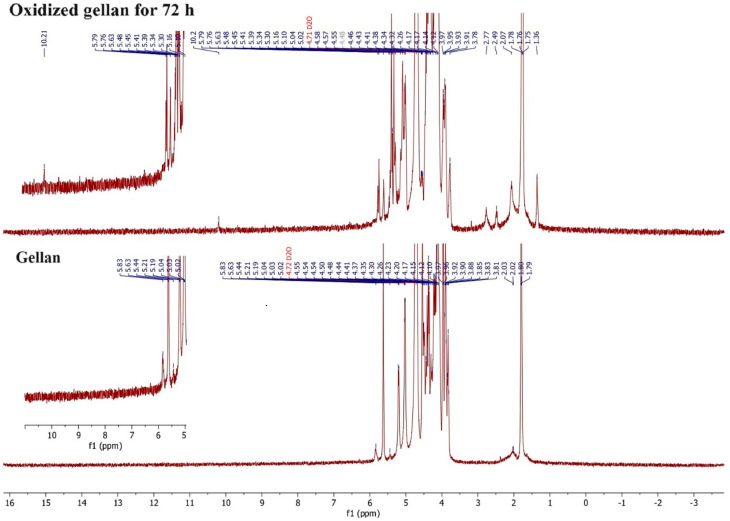
^1^H–NMR spectra recorded for standard gellan and oxidized gellan for 72 h.

A comparison was made between the ^1^H-NMR spectra of standard gellan OxG after 72 h of oxidation, as presented in Figure 8. The gellan spectrum showed clear peaks corresponding to different groups. These included the -CH_3_ group from the rhamnose unit (d 1.8 ppm); the -CH- group from glucose, glucuronic acid, and rhamnose (d 3.8–4.6 ppm); and the -CH- group belonging to glycosidic bonds in saccharides (d 5.0–5.6 ppm). The peak from the rhamnose unit, which other research mentions, was found at 1.3 ppm and was shifted at 1.8 ppm because the spectra were registered at 80 °C and not in cold deuterated water [72]. Additionally, peaks at 5.6, 5.4, and 5.2 ppm were assigned to H from C1 with glycosidic linkages of rhamnose, glucuronic acid, and glucose, respectively. In order to overcome the problem of dissolving gellan in cold deuterated water, the ^1^H-NMR spectrum of gellan was recorded at 80 °C. The results showed that the initial single peak of -CH_3_ on the rhamnose unit (around 1.8 ppm for gellan) was split into multiple peaks in the spectrum of OxG and was found to be slightly shifted at 1.75 ppm. New peaks appear in the ^1^H-NMR spectrum of OxG at 1.36 ppm, 2.49 ppm, and 2.77 ppm, which do not belong to the classical polysaccharide structures. The peak at 2.07 ppm in the OxG spectrum has a higher intensity compared to the one in the gellan spectrum. Researchers believe that some acetylate or ester groups were introduced during the oxidation process [72]. During the oxidation process of gellan in the rhamnose unit, the peaks corresponding to the -CH- glycosidic bonds, particularly those from 5.02 ppm, were observed to have smaller intensities in the OxG spectrum and at a distance slightly modified at 5.1 ppm compared to the standard gellan spectrum. The proton environment of the -CH_3_ group in the ^1^H-NMR spectrum of OxG became different due to the oxidation of the glycosidic bond in the rhamnose unit, leading to the splitting of the original peaks. These results indicate that partial cleavage of glycosidic bonds occurred during the oxidation reaction of gellan.

It was observed that a small peak occurs at 10.2 ppm in a region larger than 8.05 ppm, which belongs to aldehyde groups. The peak at 10.2 ppm indicates the presence of aldehyde groups with adjacent electrophilic groups, such as -COOH, which reduce the protective effects by decreasing the electron density of the hydrogen in aldehyde groups [73]. All the changes at the rhamnose residue indicate that the gellan oxidation reaction has occurred.

### 3.6. Determination of Free Amine Groups in Albumin Using the Ninhydrin Assay and Determination of Degree of Conversion in Hydrogel Films

**Figure 9:**
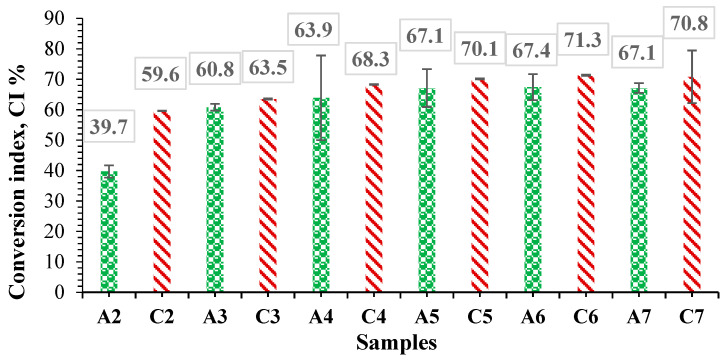
Conversion index of the amino group in Shiff bases in the presence of aldehyde groups derived from oxidized gellan for samples without inclusion complexes immobilized (samples A-green) and with inclusion complex immobilized (samples C-red).

A glutamic acid calibration curve (number of moles of NH_2_ groups in glutamic acid vs. absorbance) was plotted using the ninhydrin assay. The ninhydrin test was also used to determine amino groups in albumin solution (BSA), and based on the glutamic acid calibration curve, the number of moles of amino groups per gram of albumin was determined. The glutamic acid calibration curve had the equation y = 0.2241x (R^2^ = 0.9903). We evaluated the average number of amine groups in nine different BSA concentrations (μg/mL). The results were expressed as the number of moles of amine groups per gram of BSA, regardless of the initial concentration used. The average number of moles of amino groups per gram of BSA was 4.59 × 10^−4^ ± 0.31 × 10^−4^ moles of -NH_2_/g BSA. Hydrogel films were prepared using different molar ratios of free amine groups in BSA with aldehyde groups in OxG. The number of free -NH_2_ groups in the resulting hydrogel films was determined using the ninhydrin assay. By subtracting from the number of moles of -NH_2_ groups in BSA that were initially added in the cross-linking reaction with aldehyde groups in OxG, the number of free amine groups in the hydrogel determined using the ninhydrin test, we can determine the number of moles of -NH_2_ groups in BSA that reacted covalently with the aldehyde groups from OxG, which could form Schiff bases. Based on these results, the conversion index of amino groups in Shiff bases was determined, and Figure 9 presents the results obtained for each hydrogel film type.

It can be observed from Figure 9 that CI% increases as the number of moles of aldehyde groups from OxG increases, suggesting that a cross-linking reaction has occurred. For samples without the encapsulated inclusion complex, after the molar ratio -NH_2_:-CHO = 1:10, the CI% remains almost constant, indicating that not all amino groups in albumin react. This could be due to possible steric hindrances between macromolecules. It has been observed that the CI% value increases with the increase of the number of moles of aldehyde groups in samples containing the encapsulated curcumin–β-cyclodextrin inclusion complex. The samples containing the β-cyclodextrin–curcumin inclusion complex have a higher value of the conversion index. However, the difference between the CI% values obtained for the samples without curcumin and the CI% values for the samples containing the inclusion complex is not significant. The higher CI% values for samples with an immobilized inclusion complex may be caused by inter/intramolecular interactions such as hydrogen bonds and did not affect the cross-linking degree of the hydrogel films [47,74]. Furthermore, the inclusion of curcumin in the hydrophobic cavity of β-cyclodextrin prevents its interaction with the functional groups within BSA or OxG.

### 3.7. Determination of the Encapsulation Efficiency (Ef %)

**Figure 10:**
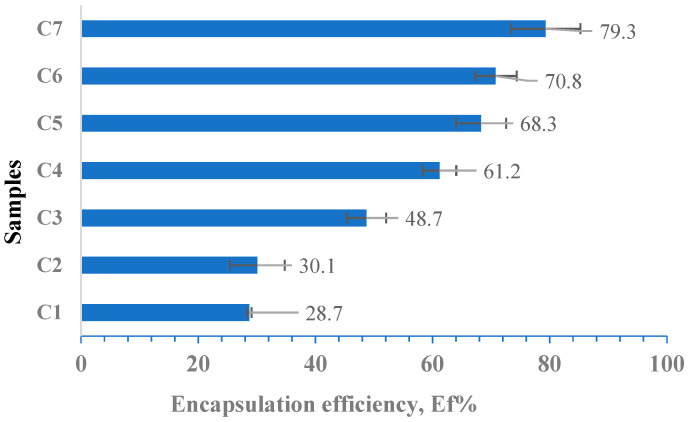
Encapsulation efficiency values for hydrogel films based on BSA cross-linked with aldehyde groups from OxG obtained at different molar ratios (Table 1).

The encapsulation efficiency of curcumin in hydrogel films was evaluated by extracting it in ethanol based on the calibration curve of curcumin in ethanol. Based on the initial amount of curcumin included in the inclusion complex immobilized (10 mg), we calculated the immobilization efficiency of polyphenol in albumin-based hydrogel films cross-linked with OxG using Equation (4). The results were obtained in triplicate and are shown as mean values in Figure 10. The equation for the calibration curve (at λ = 425 nm) is y = 0.0167x (R^2^ = 0.999).

According to Figure 10, the encapsulation efficiency for the curcumin–β-cyclodextrin inclusion complex increases as the molar ratio between the amine groups of BSA and the aldehyde groups of OxG increases. As the amount of OxG increases, the probability of interaction between the β-cyclodextrin–curcumin inclusion complex and the functional groups of biopolymers increases, which can lead to the formation of hydrogen bonds or hydrophobic interactions with the polymer matrix, thereby increasing the encapsulation efficiency. Additionally, the amount of polymer in the samples also increases when the molar ratios increase between amine groups of BSA and aldehyde groups of OxG increases, enabling the encapsulation of a more considerable amount of the β-cyclodextrin–curcumin inclusion complex, taking into account that the molecular weight of the inclusion complex is over 1 kDa.

### 3.8. FT-IR Spectroscopy for Curcumin–β-Cyclodextrin Inclusion Complex and Hydrogel Films without Immobilized Curcumin

**Figure 11:**
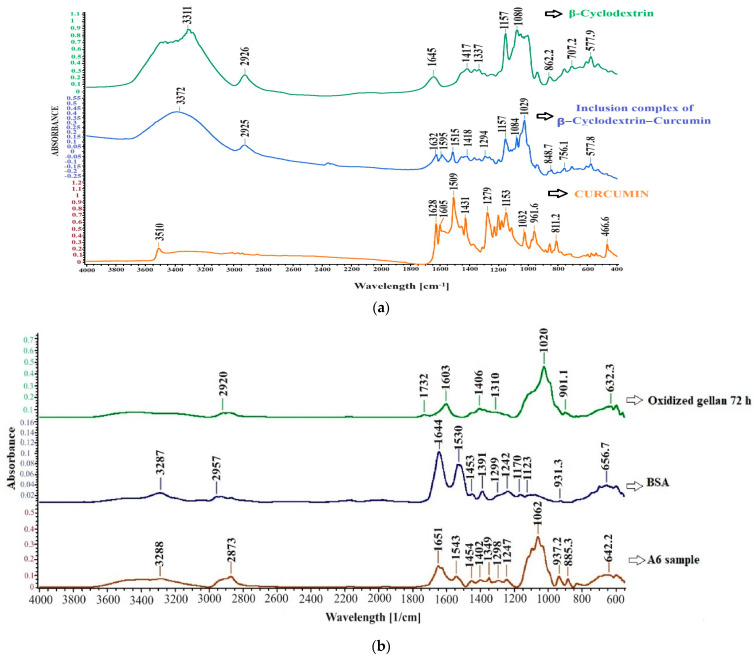
FT–IR spectra for (**a**) the inclusion complex, cyclodextrin, and curcumin, and (**b**) sample A6 (without curcumin), oxidized gellan (72 h), and albumin.

Figure 11 shows the FT-IR spectra for the inclusion complex, cyclodextrin, and curcumin in (a) and the FT-IR spectra for sample A6 (without curcumin), OxG (72 h), and albumin in (b).

In the spectrum of curcumin (Figure 11a), there were no absorption peaks in the region of carbonyl groups (1800–1650 cm^−1^), which indicates that curcumin exists in the keto-enol tautomeric form. The curcumin–β-cyclodextrin inclusion complex spectrum provided good evidence of complex formation. In the curcumin spectrum, the absorption band from 3510 cm^−1^ indicates the presence of phenolic -OH stretching vibration in curcumin. The peak from 1628 cm^−1^ indicates a stretching vibration of the benzene ring. In the spectrum of the inclusion complex, we noticed that the peak from curcumin at 3510 cm^−1^ no longer appears, providing clear evidence that the two phenolic rings of curcumin were encapsulated in the hydrophobic cavity of β-cyclodextrin. The absorption peak at 811.2 cm^−1^ from the curcumin spectrum was shifted to 848.7 cm^−1^ in the inclusion complex spectrum. Similarly, the absorption peak at 1153 cm^−1^ shifted to 1157 cm^−1^. These peaks could be assigned to the absorption band corresponding to the -C-O- vibrations, the vibrations of the functional groups in the β-cyclodextrin hydrophobic cavity.

Additionally, a shoulder in the inclusion complex spectrum is observed at 1084 cm^−1^ in this region, which may indicate complexation [75]. The characteristic band of β-cyclodextrin is found in the absorption band region between 3400 and 3300 cm^−1^ and at 2927 cm^−1^, being attributed to -OH stretching vibration and aliphatic CH or CH_2_-anisomerous stretching vibration. Multiple absorption peaks between 1030 cm^−1^ and 1080 cm^−1^ can be assigned to C-O-C vibration. In the spectrum of curcumin, there is a peak at 1279 cm^−1^ attributed to the C-O bending vibration of the OH phenolic group. However, this peak is split into several overlapping absorption peaks of lower intensities in the spectrum of the inclusion complex, suggesting that there was an interaction between β-cyclodextrin and the phenolic ring on the enol side of the curcumin molecule. In the spectrum of curcumin, there is a peak at 1509 cm^−1^ caused by the stretching vibration of the C-O group and the bending vibration of the C-C-C and C-C=O groups. In the inclusion complex β-cyclodextrin–curcumin spectrum, this peak appears to have shifted to 1515 cm^−1^. This shift provides strong evidence that complex formation has occurred. Both the C-O and C-C groups have characteristic absorption peaks in the FT-IR spectra of β-cyclodextrin and curcumin. The absorption peak at about 1605 cm^−1^ corresponds to the C-C bond. In the spectrum of the β-cyclodextrin–curcumin complex, the peak at 1605 cm^−1^ had a shoulder at 1595 cm^−1^, which indicates the formation of the inclusion complex. Therefore, the FT-IR technique successfully provided good evidence for forming the inclusion complex between β-cyclodextrin and curcumin.

The FT-IR spectra in Figure 11b show the albumin-based hydrogel films cross-linked with OxG. Albumin displays the typical amide bands specific to proteins. The peak absorption at 1644.72 cm^−1^ corresponds to the amide band’s -C=O stretching vibrations, while the absorption band at 1530 cm^−1^ represents the amide groups’ -C-N- stretching vibrations and -N-H bending vibration [76,77]. The absorption spectrum of cross-linked hydrogel films reveals a strong peak at 1651 cm^−1^, which can be attributed to the -C=N- stretching vibrations of Schiff bases and the -C=O stretching vibrations of unreacted carbonyl groups. The peak at 1543 cm^−1^ indicates a shift in the amide band, which becomes stronger after cross-linking. The spectrum of sample A6 shows peaks characteristic of OxG but they appear at slightly shifted wavelengths. However, we have observed that the peak characteristic of the aldehyde group, which is present in the spectrum of OxG at 1732 cm^−1^, is no longer detected in the spectrum of the hydrogel films, indicating the cross-linking reaction of the amine groups of BSA with the aldehyde groups in the OxG.

### 3.9. Scanning Electron Microscopy

**Figure 12:**
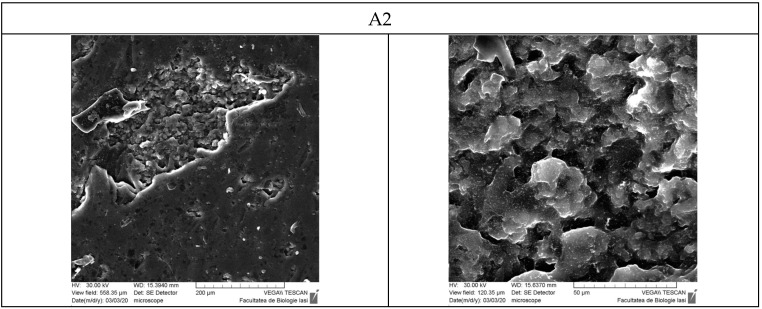

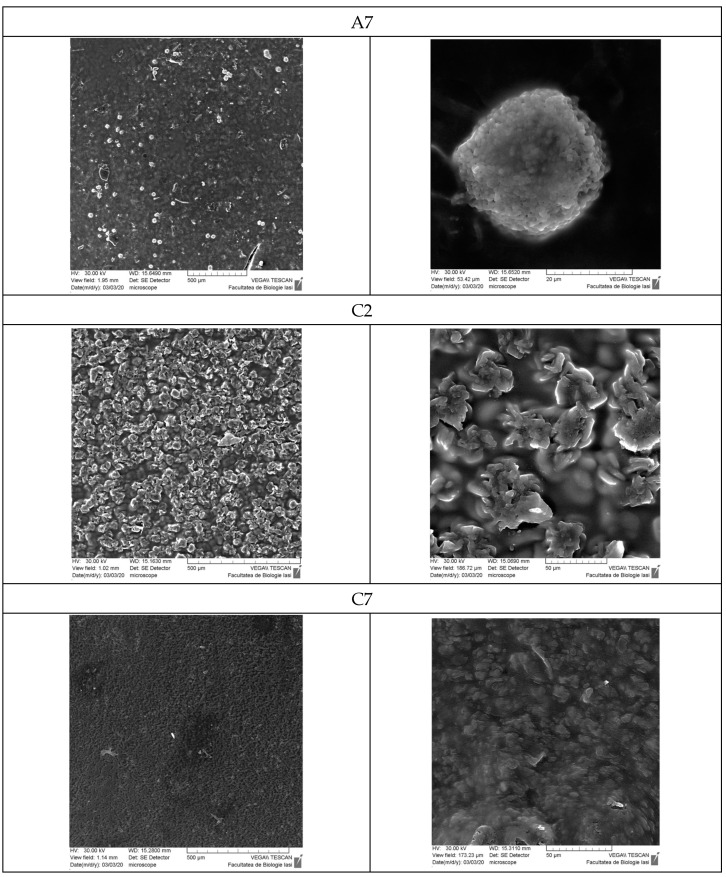
SEM photographs for hydrogel films without inclusion complex immobilized (A2 and A7 samples) and with β-cyclodextrin–curcumin inclusion complex immobilized (C2 and C7 samples).

Figure 12 presents the SEM photographs for hydrogel films without the inclusion complex immobilized (A2 and A7 samples) and with β-cyclodextrin–curcumin inclusion complex encapsulated (C2 and C7 samples).

Scanning electron microscopy was used to examine hydrogel films dried at room temperature. The section on the A2 hydrogel matrix without curcumin shows a highly porous structure with an irregular shape, containing interconnected macropores with a pore size of approximately 5 µm. Xiaoyu Ma et al. [78] obtained similar results [78]. The A2 films, which do not contain curcumin, have a smooth surface without significant porosity. The A7 film has a smooth surface; however, we observe the presence of particles, possibly caused by the precipitation of the polyelectrolyte complex between the amino groups in the albumin and the carboxylic groups in the OxG.

The reaction medium has a pH of 3, and the pKa value for the carboxylic groups is 3.5. Despite this slight difference, some of the carboxylic groups may still react with the free amino groups from the protein’s lysine residue, forming polyelectrolyte complexes. As the NH_2_/CHO molar ratio increases (see Table 1), the amount of OxG and carboxylic groups also increases, while keeping the amount of BSA constant. We can explain the close values obtained in the conversion index for samples A5, A6, and A7 based on the SEM results. The amine groups in BSA are prevented from reacting with the aldehyde groups in OxG because the polyelectrolyte complex formation occurs instantaneously, while the reaction with aldehyde groups takes time. At high temperatures (the reaction with ninhydrin takes place at 100 °C), the bonds in polyelectrolyte complexes are cleaved, leading to a greater number of free amine groups, which determines the conversion index values for samples A5, A6, and A7 to be very close. On the other hand, the C2 films, which include the β-cyclodextrin–curcumin inclusion complex, have a surface that shows significant porosity. The curcumin complex present in the film is located within the pores of the cross-linked hydrogel matrix. The curcumin complex can crystallize upon drying, forming some structures on the film surface. However, the C7 film has a much smoother surface, and the β-cyclodextrin–curcumin complex is more uniformly incorporated into the biopolymer matrix. Consequently, the complex is less likely to crystallize due to the higher amount of biopolymers in the film.

### 3.10. Determination of the Ability of Hydrogel Films to Retain Water

**Figure 13:**
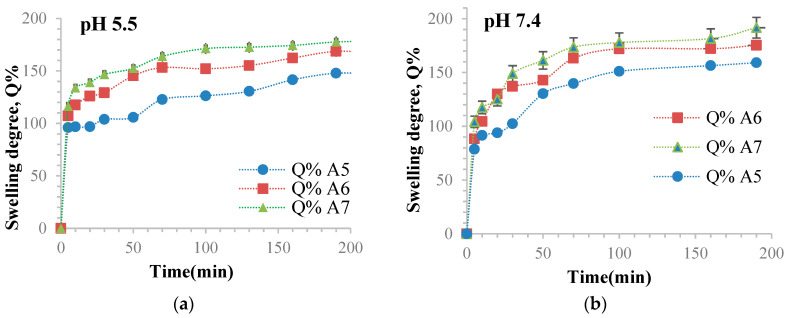
Variation of the swelling degree in time for samples A5, A6, and A7 in two different aqueous environments: (**a**) acetate buffer solution, pH = 5.5, and (**b**) phosphate buffer solution, pH = 7.4.

The hydrogel films’ swelling behavior in different aqueous media was evaluated by determining the degree of swelling (Q%) over time. This evaluation is crucial, as the degree of swelling determines the diffusion of the active principle from the films. We determined the degree of swelling (Q%) gravimetrically for samples without immobilized curcumin in two solutions: phosphate buffer solution (pH = 7.4, 0.1 M) and acetate buffer solution (pH = 5.5, 0.1 M). The results can be found in Table 2 and Figure 13.

According to Figure 13, the Q% values were slightly higher at pH = 7.4 compared to pH = 5.5 due to the presence of OxG in the hydrogel films’ polymer matrix. The basic pH causes the deprotonation of acid groups, forming carboxylate anions. This results in electrostatic repulsions between polysaccharide macromolecules, which relaxes the network and allows larger amounts of water to diffuse inside the polymer matrix [79]. It is observed that the Q% values are smaller at a pH = 5.5 than the Q% values at pH = 7.4. This happens because electrostatic repulsions are lower at pH 5.5, and the pH value of 5.5 is closer to the albumin isoelectric point, which is found between pH = 4.6 and pH = 4.9. The swelling degree of these hydrogel films mainly depends on their hydrophilicity, which is mostly determined by the presence of carboxylic groups in the OxG. In Figure 13, it can be observed that the swelling degree values increase as the amount of OxG increases due to the enhanced hydrophilicity of the hydrogel films resulting from the addition of carboxyl groups from the OxG. However, the conversion index value of amine groups to Schiff bases also increases, indicating that the degree of cross-linking is enhanced when the molar ratio between BSA and OxG is higher. The maximum swelling values were achieved due to the opposing effects of these two factors. It has been observed that the Q% value increases with the amount of OxG used in the cross-linking reaction, indicating that the hydrophilicity of the polymer network has a more significant impact than the degree of cross-linking. Therefore, it is the dominant factor that explains the results. The swelling degree was found to depend on the number of carboxyl groups in the polymer matrix, according to a research paper by Baron, R.I. et al. [80]. Their research demonstrated similar results, obtaining a physical PVA hydrogel with oxidized cellulose and oxidized pullulan [80].

### 3.11. Antioxidant Activity Determination

**Figure 14:**
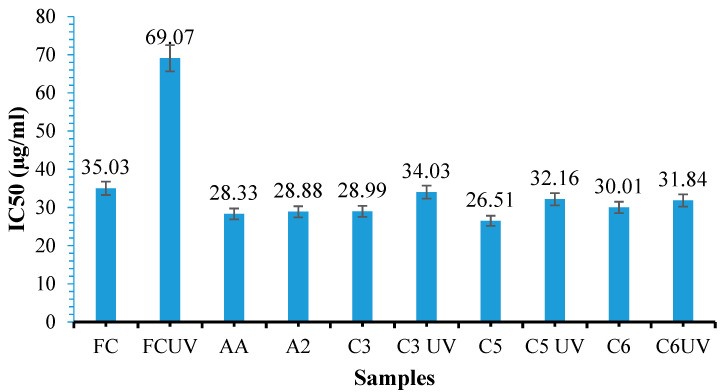
IC50 values for the antioxidant activity assay using the DPPH assay for ascorbic acid (AA), free curcumin (FC), UVA-irradiated free curcumin (FCUV), sample (A2), and for curcumin extracted from hydrogel films obtained using different molar ratios (C3, C5, C6) that were UVA-irradiated or non-UVA-irradiated.

Antioxidant activity was measured for free curcumin, UV-irradiated curcumin, ascorbic acid, and curcumin extracted from irradiated or non-irradiated hydrogel films obtained with different molar ratios between amino and aldehydic groups (C3, C5, C6). The results obtained for all the samples mentioned are presented in Figure 14. The higher the decrease in IC50 (the concentration of an antioxidant substance needed to neutralize 50% of initial DPPH radicals), the greater the antioxidant activity of curcumin. This study aimed to determine if the films can protect curcumin and be sterilized using UV light of 365 nm wavelength. The hydrogel films containing immobilized curcumin were exposed to UVA light for 30 min to test their sterilization effectiveness [81]. UVA irradiation for about 30 min can kill all pathogenic and non-pathogenic bacteria found in water [82]. The literature suggests that exposure to UVB radiation can cause a 50% degradation in curcumin [83]. Figure 14 shows the IC50 values for analyzed samples obtained after antioxidant activity evaluation.

The antioxidant activity of curcumin can be reduced due to its degradation. A recent study demonstrated that the polymer matrix protects curcumin against environmental factors that can cause degradation and reduce its bioactivity. Curcumin has three active sites that can undergo oxidation reactions through electron transfer and hydrogen abstraction. Various research groups have conducted detailed studies that confirm that when curcumin reacts with the DPPH radical, the hydrogen from the phenolic group is the most readily abstractable, leading to the formation of phenoxyl radicals [64].

Based on the findings presented in Figure 14, we can conclude that the IC50 values for ascorbic acid, free curcumin, and curcumin extracted from hydrogel films, with different molar ratios between amine and aldehyde groups, are either close to or even lower than the IC50 value for free curcumin. When free curcumin was exposed to UVA light, the IC50 value increased by 96% compared to free curcumin that had not been exposed to UVA. However, when curcumin was extracted from samples irradiated or not irradiated with UVA, we observed an increase in its antioxidant activity compared to free curcumin not irradiated with UV, as indicated by a decrease in the IC50 value. The literature mentions that curcumin could form a conjugate with albumin in the hydrophobic region (Sudlow I or II) [84].

We believe it is possible that a small amount of curcumin, which was not trapped in the hydrophobic cavity of β-cyclodextrin, interacts with the non-cross-linked albumin. As a result, the curcumin–BSA conjugate could be extracted from the film, potentially influencing the IC50 value of curcumin and leading to a slight increase in its antioxidant activity.

The antioxidant activity of the A2 hydrogel film based on BSA/OxG without curcumin was also tested. Figure 14 shows that the polymer matrix has antioxidant activity. The IC50 value depends on the molar ratio between amine and aldehyde groups used to obtain the films. For the UV-irradiated films, the IC50 value decreases with an increase in the amount of OxG in the hydrogel films, meaning that as the conversion index of amino groups into Schiff bases increases, the antioxidant activity of the curcumin incorporated in the hydrogel films also increases. A higher cross-linking degree of the hydrogel films determines better protection for curcumin included in the hydrogel film.

After extracting curcumin from UV-irradiated samples, we noticed that the IC50 value was slightly higher compared to those that were not irradiated. However, it did not exceed the IC50 value of free curcumin, leading us to the conclusion that curcumin retains its antioxidant activity through immobilization, and the polymer matrix has a protective role for curcumin against factors that can cause its degradation. The study conducted by H. Pool et al. [85] showed that curcumin, when encapsulated in PLGA-based nanoparticles, displayed increased antioxidant activity compared to free curcumin. This enhanced antioxidant activity of immobilized curcumin was also observed in another study where curcumin was encapsulated in PLGA-based nanoparticles of chitosan-chlorogenic acid conjugate [83]. Some research suggests that curcumin produces singlet oxygen and other reactive oxygen species when exposed to UV light, leading to its photodegradation [64]. Chitosan exhibits antioxidant activity and has been demonstrated to protect curcumin by donating hydrogen atoms [86].

### 3.12. Cytotoxicity Evaluation of the Films Using the MTT Assay

**Figure 15:**
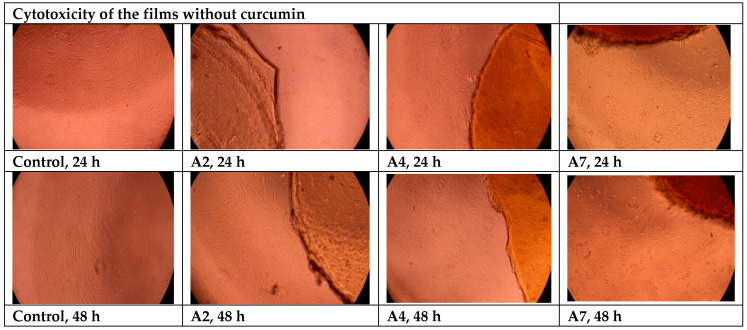
Phase contrast images of cells (fibroblast).

**Figure 16:**
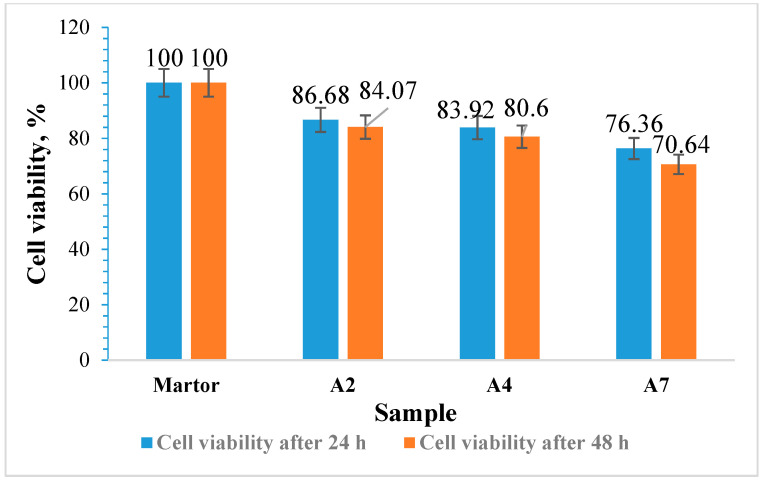
Cell viability on biopolymers films by MTT assay with normal fibroblast cells from human dermis.

The albumin-based hydrogel films were evaluated for cytotoxicity on human fibroblast cells using the MTT test over 48 h. Hydrogels A2, A4, and A7 were tested by placing them in direct contact with the fibroblast cells cultured in plates with 96 wells. The phase contrast images of cells (fibroblast) are shown in Figure 15, while Figure 16 shows the percentage of cell viability obtained after 24 and 48 h, respectively.

The results showed that the films tested A2 and A4 did not show cytotoxicity after 24 h or 48 h. The A7 hydrogel film with a molar ratio of 16:1 between the aldehyde groups in the OxG and the amine groups in the albumin showed slight toxicity, with cell viability being 76.6% after 24 h and 70.64% after 48 h. We have observed a decrease in cell viability for all samples analyzed after 48 h. Moreover, cell viability has been found to decrease with an increase in the -NH_2_/-CHO molar ratio. These results are consistent with previous studies that explored cytotoxicity in oxidized polysaccharides like oxidized alginate and oxidized hyaluronic acid [87].

The cytotoxic effects observed are due to the presence of the free reactive aldehyde groups that are generated during the oxidation process. These aldehydic groups can interact with primary amine functional groups present in the protein of cell membranes of biological systems, thereby causing damage to the cells [88]. The observed cytotoxicity of OxG after 48 h may be due to its relatively low molecular weight (Mw). A lower molecular weight increases the availability and mobility of reactive aldehyde groups. As a result, fewer intramolecular hydrogen bonds can be formed, allowing the reactive aldehyde groups easier access to cells [89].

### 3.13. Curcumin Release Kinetics

This study investigated the release kinetics of curcumin in two pH media, namely, phosphate buffer 0.1 M pH = 7.4 and acetate buffer 0.1 M pH = 5.5, at physiological temperature (T = 37 °C) for 24 h.

**Figure 17:**
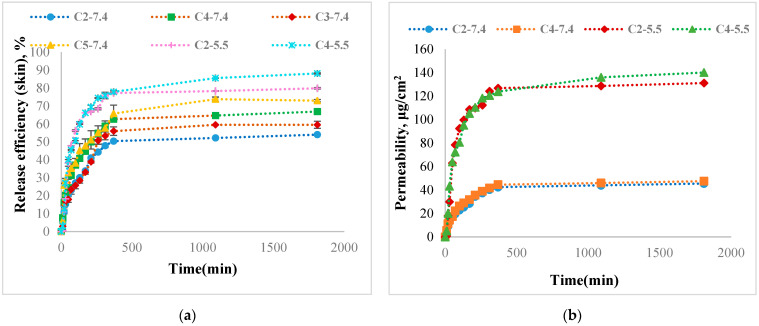
The curcumin release kinetics through the skin using a Franz cell in time in 0.1 M acetate buffer solution at pH = 5.5 for C2 and C4 samples and in 0.1 M phosphate buffer solution at pH 7.4 for C2, C3, C4, and C5 samples expressed as release efficiency (**a**) or in terms of permeability μg/cm^2^ (**b**) for samples C2 and C4 for both pH solutions.

The release kinetics of curcumin from the obtained films were studied using a Franz cell and chicken skin as a membrane. This method has been described in the characterization methods section. Figure 17 displays the curcumin release curves in 0.1 M acetate buffer solution at pH = 5.5 containing 1% Tween 80 for samples C2 and C4 and in a 0.1 M phosphate buffer solution containing 1% Tween 80 at pH = 7.4 for samples C2, C3, C4, and C5. These samples were selected to clearly show the molar ratio’s influence -CHO/-NH_2_ on the process of curcumin release from the films. The surfactant Tween 80 was used in the release medium at a concentration of 1% because curcumin, which is hydrophobic, is not soluble in water. The use of Tween 80 helps prevent the precipitation and degradation of curcumin after release, as curcumin is known to degrade rapidly at pH 7.4 [90,91]. Calibration curves were plotted using various concentrations of curcumin in buffer solutions with different pH levels, to which 1% Tween was added. In Figure 17b, the curcumin permeability through the skin membrane was calculated based on the curcumin released in the receptor compartment of the Franz cell, and the curcumin within the chicken skin membrane was not considered. Table 3 shows the release efficiency and permeability values for the immobilized inclusion complex of β-cyclodextrin–curcumin through the membrane in the Franz cell’s receptor compartment. The exponential factor n calculated from the Ritger–Peppas equation is also presented in Table 3.

Figure 17a presents the curves that represent the release of curcumin after approximately 24 h at two different pH values—5.5 and 7.4. The release curves clearly show that there was no initial burst release at either pH. Instead, a controlled release was consistently observed over time, indicating the homogeneous encapsulation of curcumin in the hydrogel films. The initial burst release of the drug from nanoparticles generally occurs due to the deposition of the drug on the surface of the nanoparticles, often due to inadequate encapsulation [92]. According to Table 3 and Figure 17, the release efficiency of curcumin from the receptor compartment was higher at pH = 5.5 than at pH = 7.4. The samples obtained at a higher molar report had the highest release efficiency value, indicating that irrespective of the pH value, the release efficiency increases with the amount of OxG, increasing the samples’ hydrophilicity. Moreover, it was observed that the amount of curcumin retained by the membrane at pH = 7.4 was lower than that of curcumin retained at pH = 5.5, as shown in Table 3, as permeability in the skin membrane (μg/cm^2^/h).

Figure 17b displays the time-dependent changes in the curcumin permeability in the receptor compartment expressed in µg/cm^2^ for 24 h for samples C2 and C4 at pH values of 7.4 and 5.5. We have only presented data for these two samples because their permeability values are very similar at pH = 7.4.

The release efficiency of curcumin at pH = 7.4 depends on the level of hydrophilicity in the samples, meaning that the amount of OxG in the samples and, consequently, the amount of carboxylic groups in the films affect the release. Additionally, curcumin dissolves more easily in slightly alkaline pH levels than in acidic ones.

At pH = 5.5, the amine groups from proteins within the skin membrane can become protonated and interact with the free aldehyde groups present in the films [93,94], meaning that hydrogel films containing more free aldehyde groups can attach to the skin membrane more efficiently and release curcumin with greater effectiveness. In a previous research paper, it was demonstrated that curcumin can be released in a controlled manner through chicken skin from hydrogel films based on chitosan and oxidized carboxymethyl cellulose (CMCOx) [51]. This paper’s research observed that the encapsulated curcumin can be released through the skin with higher efficiency when the pH of the release medium is 5.5. The total skin permeability at pH = 5.5 had values between 2.27 μg/cm^2^/h and 3.3 μg/cm^2^/h, and at pH = 7.4, the values were between 1.33 μg/cm^2^/h and 2.21 μg/cm^2^/h. This study found that chitosan films cross-linked with CMCOx had higher curcumin permeability through the skin. This was due to the bioadhesive properties of chitosan and the interaction between the carboxylic groups of the oxidized polysaccharide and the lysine residue in the skin protein.

Moreover, hydrogel films based on BSA cross-linked with OxG had higher total permeability at pH 5.5 compared to pH 7.4. The total permeability values obtained are higher than those obtained for curcumin released from chitosan/CMCOx films due to the higher hydrophilicity of the BSA/OxG hydrogel films, which was determined by the higher number of carboxylic groups in the OxG and the fact that the curcumin–β-cyclodextrin inclusion complex used to increase the solubility of curcumin in aqueous solutions was encapsulated in the hydrogel films. The amount of free carboxylic and aldehyde groups was higher compared with the case of chitosan/CMCOx films because higher -NH_2_/-CHO molar ratios of up to 1/16, were used to prepare the hydrogels, which cause stronger interactions with skin proteins that contains residues of lysine. Albumin also has bioadhesive properties and can attach to the skin membrane. The permeability values at pH = 5.5 obtained for the C2 hydrogel film were 5.77 μg/cm^2^/h, and for the C4 hydrogel film, it was 6.35 μg/cm^2^/h. At pH = 7.4, the total permeability values were between 1.99 μg/cm^2^/h for sample C2 and 2.23 μg/cm^2^/h for sample C5. The literature mentions different types of delivery systems for evaluation using Franz cells of curcumin permeability for topical applications. Table 4 presents the in vitro permeability evaluation of curcumin encapsulated in other delivery systems using the Franz cell.

Table 4 illustrates that the release of curcumin from various release systems depends on the type of release system utilized (microemulsion, nanoemulsion, nanofibers, or nanoparticles), the release medium employed, the type of membrane used, and the quantity of immobilized curcumin. In general, curcumin released from micro/nanoemulsions has higher permeability than curcumin released from hydrogels. The main disadvantage of using such systems is that the release of curcumin is not controlled and sustained over a longer period of time, and the system used does not have the ability to absorb the exudate from different wounds. Ethosomes have a drawback in that the loading efficiency of curcumin is low, and these release systems are not very stable over time. However, they do have the ability to diffuse into several layers of the skin. Transdermal delivery of curcumin from polymer-based delivery systems is lower compared to micro/nanoemulsions, possibly due to the low amount of encapsulated curcumin. Polymeric delivery systems are typically hydrophilic, while curcumin is a hydrophobic polyphenol. This leads to low encapsulation efficiency and, consequently, low permeability. Compared to other polymer-based delivery systems, we have observed that BSA/OxG-based hydrogel films exhibit comparable or even superior skin permeability of curcumin compared to other delivery systems. The improved permeability may be attributed to the albumin containing a hydrophobic region that can interact with the polyphenol, leading to higher encapsulation efficiency. Additionally, the β-cyclodextrin–curcumin inclusion complex increases the solubility of curcumin. The hydrogel film adheres to the skin membrane due to the presence of free aldehydic groups from OxG, which can interact with the amine groups of skin proteins. We believe that the obtained delivery system based on BSA/OxG with encapsulated curcumin can be successfully used to treat various skin conditions.

Using the Ritger–Peppas kinetic model can provide more information on how curcumin is transported and released from the polymer matrix [101]:

(11) $$\frac{M_{t}}{M_{\infty }}\;=\;k\;*\;t^{n}$$

where M_t_ is defined as the mass of drugs released at time t, $M_{\infty }$—the mass of the drug released as the time approaches infinity, k is a constant, and n is the diffusional exponent.

Based on the curcumin release curves, the exponential factor n value determined using the Ritger–Peppas equation indicated that the diffusion mechanism was mainly Fickian, being equal to 0.5 or lower for most of the samples used to study the release kinetics (the release mechanism is conducted by diffusion). However, for the curcumin release from the C2 sample at pH = 5.5, the exponential factor n value was 0.6, indicating a non-Fickian type of diffusion mechanism that was possibly affected by certain factors that have not been studied yet (possibly diffusion and degradation mechanism). Fickian diffusion occurs when the polymer’s (tr) relaxation time exceeds the solvent’s (td) diffusion time. Non-Fickian diffusion, on the other hand, occurs when tr ≈ td and cannot be described by Fick’s laws of diffusion [102].

On the other hand, for curcumin release from the C5 sample at pH = 7.4, the exponential factor n value was 0.3, indicating a semi-Fickian type of diffusion mechanism that was likely disturbed by interactions between curcumin and the functional groups of polymers from hydrogels. Additionally, it was observed that at pH = 7.4, the exponential factor n decreased when the quantity of OxG increased, suggesting possible interactions, such as hydrogen bonds or hydrophobic interactions between the drug and the polymer matrix that increase when the aldehyde or carboxylic groups within the polymer matrix increase. In conclusion, the release of curcumin from BSA/OxGO films is governed by diffusion.

## 4. Conclusions

Gellan gum was oxidized using sodium periodate, and its structure was analyzed using FT-IR and ^1^H-NMR spectroscopy. The oxidation kinetics and molecular weight were evaluated. The results indicate that the maximum degree of oxidation was achieved after 117 h of oxidation, with a value of 57.16%. Additionally, it was observed that the molecular weight of the OxG decreased as the oxidation time increased. FT-IR spectroscopy has confirmed the presence of aldehyde groups within the OxG, and the ^1^H-NMR spectroscopy shows good evidence that the oxidation of gellan in the presence of NaIO_4_ occurs in rhamnose residue. Hydrogel films were prepared using albumin and OxG in different molar ratios between the amine groups in albumin and the aldehyde groups in OxG. The results revealed that the conversion index increased as the amount of OxG in the hydrogel film increased. The FT-IR spectrum of the hydrogel film shows that Schiff bases were formed by cross-linking between the amino groups of albumin and the carbonyl groups of OxG.

The swelling degree was measured for the hydrogel films obtained, and it was observed that its values depended on the crosslinking degree but mainly the hydrophilicity of the samples. The swelling degree of the polymer matrix increases as the number of carboxyl groups increases due to the increase in OxG within the hydrogel films. The value of the swelling degree is influenced by pH, which was found to be higher at pH = 7.4 compared to pH = 5.5. This difference in swelling degree values is determined by the presence of carboxyl groups in the film. Cytotoxicity of the hydrogel films was evaluated using the MTT assay, and it was shown that hydrogel films with -CHO:-NH_2_ molar ratios up to 16:1 did not show cytotoxic effects. Cell viability decreases as the number of moles of free carbonyl groups in the film increases. However, except for the 16:1 molar ratio sample, which has a slight cytotoxic effect, the hydrogel films did not show cytotoxicity and can be used as biocompatible biomaterials.

Antioxidant activity was tested for free curcumin, free curcumin irradiated with UVA light for 30 min, curcumin extracted from UVA-irradiated or non-UVA-irradiated hydrogel films, and hydrogel films without curcumin. The IC50 value decreased with the increase in the amount of OxG in the hydrogel films. In all the analyzed cases, the antioxidant activity of curcumin extracted from films irradiated or not irradiated with UVA is superior to that of free curcumin or free curcumin irradiated with UVA, the polymer matrix having a protective role for curcumin. The release of curcumin from the films was tested on chicken skin using a Franz cell. The results showed a higher release efficiency on chicken skin at pH = 5.5 compared to pH = 7.4 because the free aldehyde and carboxylic groups can interact with the amine groups in the skin used as the membrane, and the film attaches and more efficiently releases curcumin through the skin.

## Figures and Tables

**Table 1:** The experimental program for obtaining hydrogels based on BSA and oxidized gellan.

Sample Code *	Molar Report, -NH_2_/-CHO	Number of -NH_2_ Moles from BSA	Number of -CHO Moles from Oxidized Gellan	Amount of Curcumin within the Inclusion Complex (mg)
A1	1:1	4.59 × 10^−5^	4.59 × 10^−5^	-
A2	1:4		1.84 × 10^−4^	
A3	1:6		2.75 × 10^−4^	
A4	1:8		3.67 × 10^−4^	
A5	1:10		4.59 × 10^−4^	
A6	1:12		5.51 × 10^−4^	
A7	1:16		7.34 × 10^−4^	
C1	1:1		4.59 × 10^−4^	10
C2	1:4		1.84 × 10^−4^	
C3	1:6		2.75 × 10^−4^	
C4	1:8		3.67 × 10^−4^	
C5	1:10		4.59 × 10^−4^	
C6	1:12		5.51 × 10^−4^	
C7	1:16		7.34 × 10^−4^	

* 5 mL of solution at pH = 3, 0.1 M, and 1% (*w*/*w*) tetraethylene glycol were used. The same oxidized gellan was used for all the samples, and the quantity of oxidized polysaccharides increased when the molar report between amino and aldehyde groups increased. The number of moles of amino groups was maintained constantly.

**Table 2:** Results obtained for maximum (Q%) values for samples without curcumin were obtained after 190 min.

Sample Code	The Maximum Value of the Swelling Degree (Q%) in ABS, at pH = 5.5	The Maximum Value of the Swelling Degree (Q%) in PBS, at pH = 7.4
A1	23.81	73.52
A2	57.73	77.23
A3	100.7	128.53
A4	105.54	134.04
A5	147.78	159.11
A6	166.56	175.28
A7	178.28	191.64

**Table 3:** The permeability and release efficiency of curcumin-loaded hydrogel films through the skin membrane.

Sample Code	Release Efficiency after 24 h, %	The Permeability Coefficient of CURC Found in the Receptor Compartment after 24 h, μg/cm^2^/h	Curcumin Permeability Coefficient in the Skin Membrane, μg/cm^2^/h	Total Permeability Coefficient, μg/cm^2^/h	Exponential Factor, n	R^2^
C2-7.4	54.03 ± 0.14	1.9 ± 0.005	0.09	1.99	0.5	0.9151
C3-7.4	59.52 ± 1.95	1.9 ± 0.08	0.1	2	0.48	0.9557
C4-7.4	66.9 ± 1.1	2 ± 0.05	0.13	2.13	0.47	0.9949
C5-7.4	73 ± 0.64	2.1 ± 0.03	0.13	2.26	0.3	0.9893
C2-5.5	79.9 ± 0.3	5.5 ± 0.01	0.27	5.77	0.6	0.8547
C4-5.5	88.13 ± 0.4	5.83 ± 0.016	0.52	6.35	0.4	0.9607

**Table 4:** Evaluation of curcumin permeability through the skin in vitro from different drug delivery systems using Franz cell.

Nr. Crt	Curcumin Release System Type	Medium Used in the Receptor Compartment	Dimension	Membrane Type	Permeability μg/cm^2^/h	Ref.
1	Stable microemulsions of curcumin using different oils and surfactants	PBS and ethanol 1:1	Droplet diameter 199.39 ± 0.017	Mouse skin	130.91 ± 0.02	[95]
2	Microemulsion-based keratin–chitosan gel with encapsulated curcumin	PBS with 1% Tween	The particle size of the CME-KCS gel was 186.45 ± 0.75 nm	Rat skin	0.16 ± 0.01	[96]
3	Curcumin-loaded cellulose acetate phthalate nonwoven electrospun nanofiber	Phosphate buffer at pH = 7.4 with 20% ethanol	300 nm	Pig abdominal skin	12.87	[97]
4	Nanoemulsion based on β-lactoglobulin with curcumin-encapsulated	90/10 (*v*/*v*) PB/ethanol, 0.1 wt% Tween 20, and 0.04 wt% ascorbic acid	220 nm	Synthetic membrane	0.47	[98]
5	Chitosan nanoparticles with curcumin encapsulated	PBS. pH = 7.4 with 1% Tween 80	167.3 ± 3.8 nm–251.5 ± 5.8 nm	Strat-M membrane made of polyester sulfone	0.54 ± 0.03 and 0.44 ± 0.03	[92]
6	Ethosomes (ETs) with encapsulated curcumin	Ethanol:water mixture (50:50, *v*/*v*)	200 nm	STRAT-M^®^ membranes	0.70 ± 0.21	[99]
7	Microneedles containing hyaluronidase with nano curcumin encapsulated	PEG 400 aqueous solution was used as a receptor medium	55 nm	Pig skin	From 0.68 ± 0.16 to 2.79 ± 0.20	[100]

## Data Availability

The data presented in this study are available upon request from the corresponding author.
